# An early bothremydid from the Arlington Archosaur Site of Texas

**DOI:** 10.1038/s41598-021-88905-1

**Published:** 2021-05-20

**Authors:** Brent Adrian, Heather F. Smith, Christopher R. Noto, Aryeh Grossman

**Affiliations:** 1 Department of Anatomy, Midwestern University, Glendale, AZ USA; 2 Department of Biological Sciences, University of Wisconsin-Parkside, Kenosha, WI USA

**Keywords:** Palaeontology, Phylogenetics

## Abstract

Four turtle taxa are previously documented from the Cenomanian Arlington Archosaur Site (AAS) of the Lewisville Formation (Woodbine Group) in Texas. Herein, we describe a new side-necked turtle (Pleurodira), *Pleurochayah appalachius* gen. et sp. nov., which is a basal member of the Bothremydidae. *Pleurochayah appalachius* gen. et sp. nov. shares synapomorphic characters with other bothremydids, including shared traits with Kurmademydini and Cearachelyini, but has a unique combination of skull and shell traits. The new taxon is significant because it is the oldest crown pleurodiran turtle from North America and Laurasia, predating bothremynines *Algorachelus peregrinus* and *Paiutemys tibert* from Europe and North America respectively. This discovery also documents the oldest evidence of dispersal of crown Pleurodira from Gondwana to Laurasia. *Pleurochayah appalachius* gen. et sp. nov. is compared to previously described fossil pleurodires, placed in a modified phylogenetic analysis of pelomedusoid turtles, and discussed in the context of pleurodiran distribution in the mid-Cretaceous. Its unique combination of characters demonstrates marine adaptation and dispersal capability among basal bothremydids.

## Introduction

Pleurodira, colloquially known as “side-necked” turtles, form one of two major clades of turtles known from the Early Cretaceous to present^1,2^. Pleurodires are Gondwanan in origin, with the oldest unambiguous crown pleurodire dated to the Barremian in the Early Cretaceous^2^. Pleurodiran fossils typically come from relatively warm regions, and have a more limited distribution than Cryptodira (hidden-neck turtles)^3–6^. Living pleurodires are restricted to tropical regions once belonging to Gondwana^7,8^. The separation of Laurasia and Gondwana and subsequent fragmentation of their landmasses in the Early Cretaceous, along with variations in global temperatures, affected the distribution of some pleurodire lineages^3,9^.

One pleurodiran lineage, Bothremydodda (Taphrosphyini + Bothremydini, sensu Gaffney et al*.*^10^) is the only published pleurodiran lineage to disperse from Gondwana to Laurasia in the Cretaceous^5^. The oldest genus of Bothremydidae found in Laurasian deposits is *Algorachelus*^8^. *Algorachelus peregrinus* is found in coastal environments at the southern Laurasian middle-late Cenomanian sites of Algora, Guadalajara, Castile and Leon, Spain and Nazaré, Portugal^8,9,11^. *Algorachelus parvus* is represented in the early or middle Cenomanian of Palestine^12^. Finally, *Paiutemys tibert* is known from brackish uppermost Cenomanian deposits of the Laramidian Naturitas Formation in Utah, USA^9,13^. While some authors have subsumed *P. tibert* within *Algorachelus*^9^, Hermanson et al.^14^ did not support the position of *Paiutemys tibert* within the genus *Algorachelus*. *Paiutemys tibert* is not closely related to any other North American bothremydid, and represents a first and independent dispersal of Bothremydini to the continent^9,13^. Thus, including the new taxon described here, Bothremydidae from Gondwana reached the east coast of the Atlantic Ocean and Appalachia at least in the middle Cenomanian, and Laramidia at least in the late Cenomanian.

Here, we describe *Pleurochayah appalachius*, gen. et sp. nov. (Pleurodira: Bothremydidae), from the Arlington Archosaur Site—the second and earliest pleurodiran lineage found in Laurasian deposits. *Pleurochayah appalachius* gen. et sp. nov. has a unique combination of characters, including many shared with Kurmademydini and Cearachelyini, as well as some traits shared with later derived bothremydids, Bothremydini and Taphrosphyini. In addition to cranial, shell, and postcranial anatomy, we also describe and analyze the shell microanatomy of *Pleurochayah appalachius* gen. et sp. nov. and compare it to histology from related taxa.

## Geological setting

**Figure 1:**
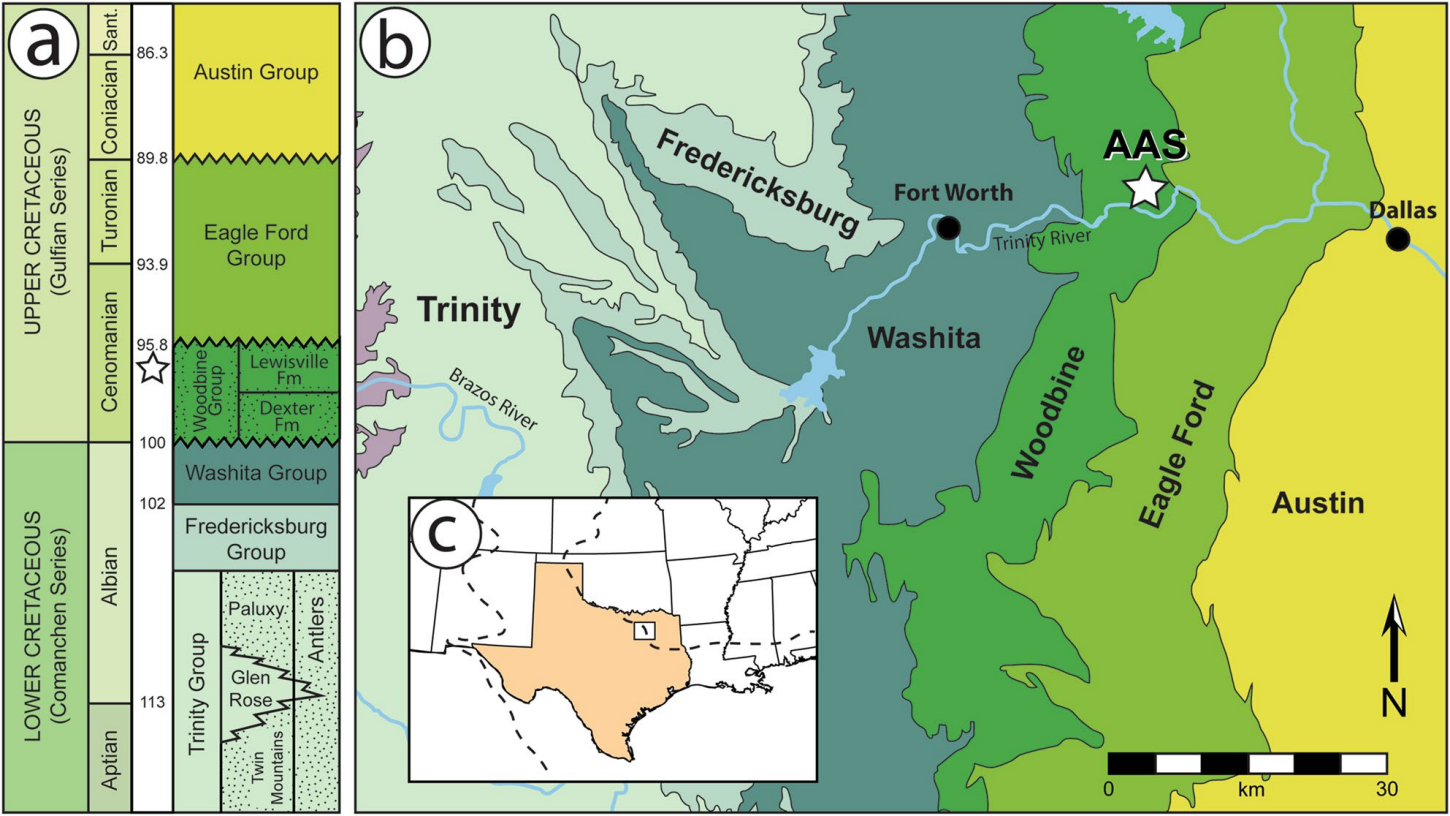
Location and geologic setting of the Arlington Archosaur Site (AAS marked with star). (**a**) Stratigraphic column and selected ages of Cretaceous rock units in north-central Texas, using stratigraphic nomenclature for the Woodbine Gp from Denne et al.^26^. Terrestrial deposits indicated by stippled patterns. Modified from Winkler et al.^27^ and Jacobs et al.^28^; (**b**) Generalized map of geologic units in the Fort Worth Basin; (**c**) Inset map showing approximate location of the AAS in relation to the southern Western Interior Seaway [WIS] (modified after Barnes et al.^29^ and Strganac^30^). Approximate WIS shorelines during the Cenomanian are dotted (after Blakey^31^). Figure created with Photoshop CS5 (Adobe Inc.).

The fossils derive from the Arlington Archosaur Site (AAS) in sediments most likely deposited near the middle of the Lewisville Formation [Fm] of the Upper Cretaceous Woodbine Group. The Woodbine Group [Gp] (lower middle Cenomanian) stretches from Temple in central Texas to Lake Texoma in southern Oklahoma, and is the oldest known unit in the Gulf Coastal Plain^15–21^ (Fig. 1a). The Woodbine Group sits unconformably between the older Grayson Marl of the Washita Group and the younger Eagle Ford Group^17,18,20^ (Fig. 1). At least ten million years of marine deposition separate the Woodbine Gp from the earlier terrestrial depositional systems of the Lower Cretaceous Trinity Gp^22^ (Fig. 1a). The minimum age for the of Upper Woodbine Gp and Tarrant Fm of the lower Eagle Ford Gp is 95–96 Ma, established by presence of *Conlinoceras tarrantense*, a zonal marker for the base of the middle Cenomanian^20,21,23–25^. The Woodbine Group comprises two Formations: the Dexter Fm overlain by the Lewisville Fm^26^ (Fig. 1a). The four lithologic units comprising the Lewisville Fm, from oldest to youngest are the Red Branch, an unnamed interval, Arlington, and Tarrant Members [Mbrs]^26^. The AAS deposits containing the fossils described herein most likely lie in the interval between the Arlington and marine Red Branch Mbrs^26^. All specimens described herein were found unassociated. See “Supplementary Information” for further geological background.

## Results

### Systematic paleontology

Pleurodira Cope, 1865^32^.

Pelomedusoides Broin, 1988^3^.

Bothremydidae Baur, 1891^33^.

*Pleurochayah* LSID: urn:lsid:zoobank.org:act:5D12B06D-9E3F-496C-A842-585F73AFAD68 gen. nov.

**Figure 2:**
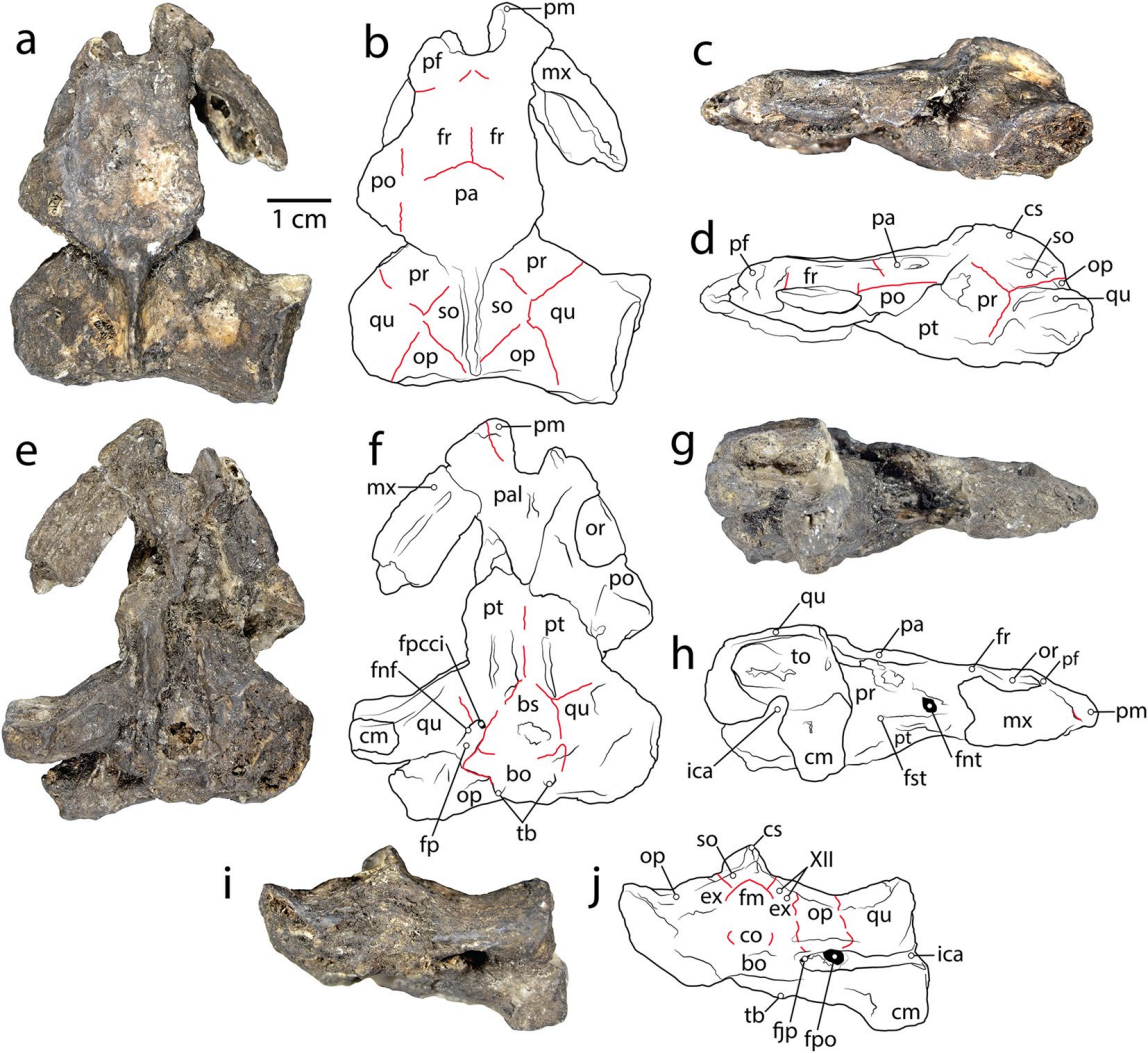
DMNH 2013-07-1782, a partial skull and type specimen of *Pleurochayah appalachius* gen. et sp. nov. (**a**) Dorsal photograph, (**b**) dorsal drawing, (**c**) left lateral photograph, (**d**) left lateral drawing, (**e**) ventral photograph, (**f**) ventral drawing, (**g**) right lateral photograph, (**h**) right lateral drawing, (**i**) posterior photograph, and (**j**) posterior drawing. *bo* basioccipital, *bs* basisphenoid, *cm* condylus mandibularis, *co* condylus occipitalis, *cs* crista supraoccipitalis, *ex* exoccipital, *fjp* foramen jugulare posterius, *fnt* foramen nervi trigeminale, *fp* fossa pterygoidea, *fpcci* foramen posterius canalis caroticus internus, *fr* frontal, *fst* foramen stapediotemporale, *ica* incisura columella auris, *iof* interorbital foramen, *mx* maxilla, *op* opisthotic, *or* orbit, *pa* parietal, *pal* palatine, *pf* prefrontal, *pm* premaxilla, *po* postorbital, *pr* prootic, *pt* pterygoid, *qu* quadrate, *so* supraoccipital, *sq* squamosal, *to* tympanic opening, *tb* tuberculum basioccipitale, *XII* nervi hypoglossi. Red lines indicate visible sutures. Figure created with Adobe Creative Cloud (Adobe Inc.).

**Figure 3:**
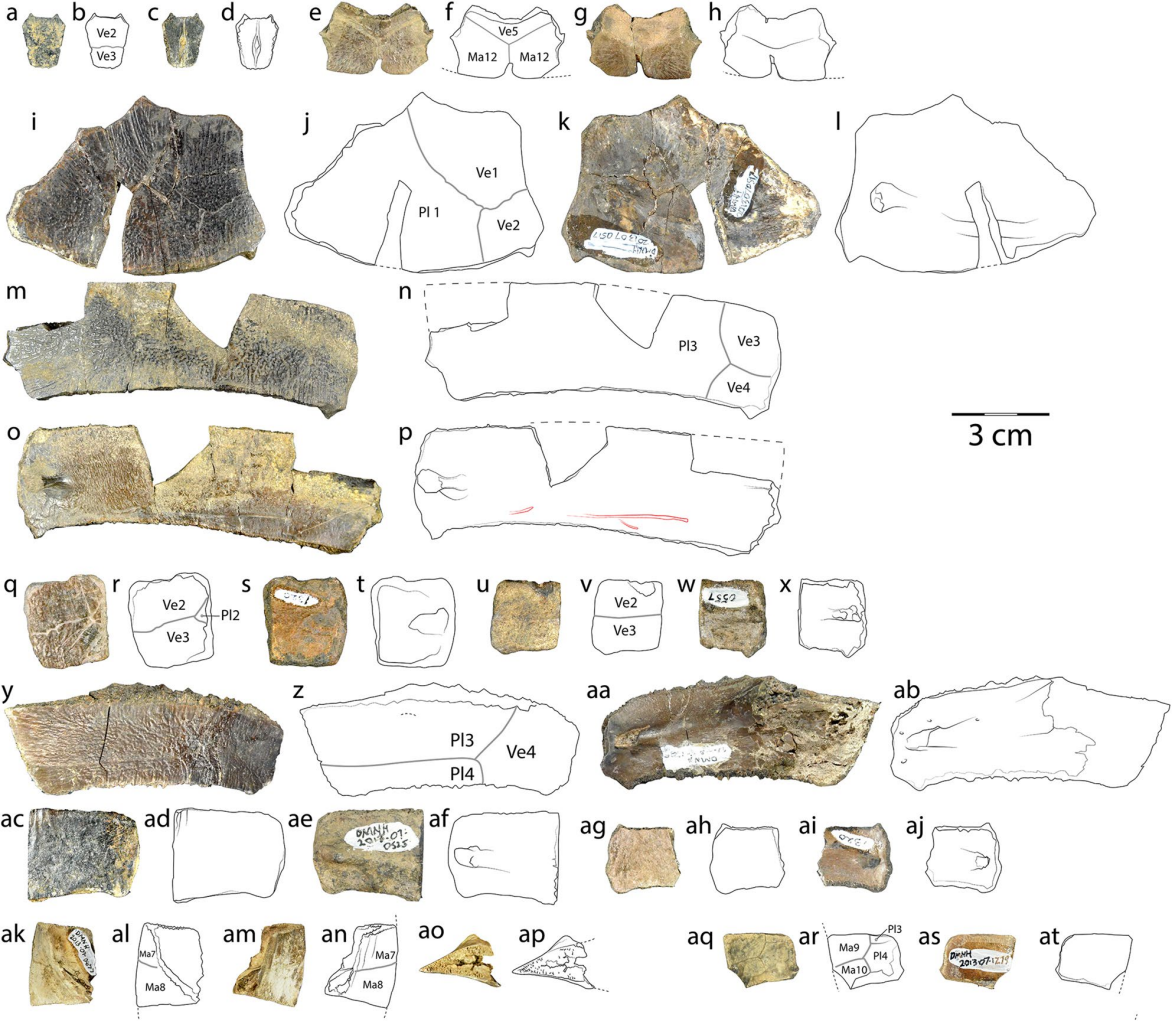
Carapacial specimens of *Pleurochayah appalachius* gen. et sp. nov., from the Cenomanian (Upper Cretaceous) Arlington Archosaur Site (Lewisville Fm, Woodbine Gp). (**a**) Dorsal photograph, (**b**) dorsal drawing, (**c**) ventral photograph, and (**d**) ventral drawing of DMNH 2013-07-0683, neural 3. (**e**) Dorsal photograph, (**f**) dorsal drawing, (**g**) ventral photograph, and (**h**) ventral drawing of DMNH 2013-07-1998, pygal. (**i**) Dorsal photograph, (**j**) dorsal drawing, (**k**) ventral photograph, and (**l**) ventral drawing of DMNH 2013-07-0517, left costal 1. (**m**) Dorsal photograph, (**n**) dorsal drawing, (**o**) ventral photograph, and (**p**) ventral drawing of DMNH 2013-07-1999, left costal 5. (**q**) Dorsal photograph, (**r**) dorsal drawing, (**s**) ventral photograph, and (**t**) ventral drawing of DMNH 2013-07-1320, partial cf. right costal 3. (**u**) Dorsal photograph, (**v**) dorsal drawing, (**w**) ventral photograph, and (**x**) ventral drawing of DMNH 2013-07-0557, partial cf. right costal 3. (**y**) Dorsal photograph, (**z**) dorsal drawing, (**aa**) ventral photograph, and (**ab**) ventral drawing of DMNH 2013-07-1405, left costal 6. (**ac**) Dorsal photograph, (**ad**) dorsal drawing, (**ae**) ventral photograph, and (**af**) ventral drawing of DMNH 2013-07-0525, partial cf. left costal 4. (**ag**) Dorsal photograph, (**ah**) dorsal drawing, (**ai**) ventral photograph, and (**aj**) ventral drawing of DMNH 2013-07-1320, partial cf. right costal 4. (**ak**) Dorsal photograph, (**al**) dorsal drawing, (**am**) ventral photograph, (**an**) ventral drawing, (**ao**) posterior photograph, and (**ap**) posterior drawing of DMNH 2013-07-0673, right peripheral 7. (**aq**) Dorsal photograph, (**ar**) dorsal drawing, (**as**) ventral photograph, and (**at**) ventral drawing of DMNH 2013-07-1279, left peripheral 9. All parts of figure to same scale. Figure created with Adobe Creative Cloud (Adobe Inc.).

**Figure 4:**
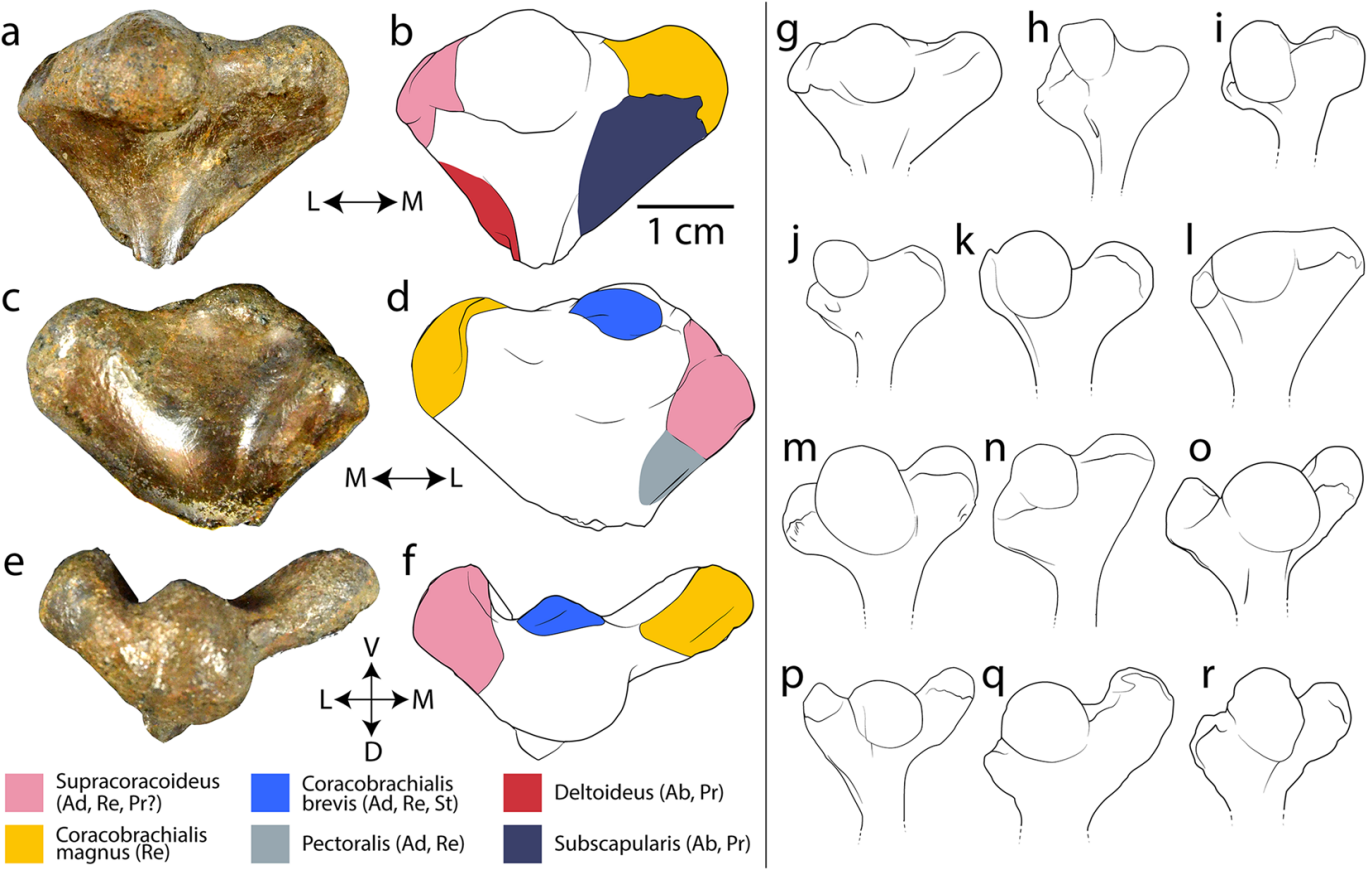
DMNH-2013-07-0500, an isolated proximal left humerus of *Pleurochayah appalachius* gen. et sp. nov. with inferred muscle attachments. (**a**) Dorsal photograph, (**b**) dorsal drawing, (**c**) ventral photograph, (**d**) ventral drawing, (**e**) proximal photograph, and (**f**) proximal drawing. Interpretations of muscle attachments based on Walker^34^ and Krahl et al.^35^. Comparison of the proximal humerus (dorsal views) of fossil and extant pleurodires: (**g**) *Pleurochayah appalachius* gen. et sp. nov., (**h**) *Podocnemis alabamae*^36^, (**i**) *Bothremys* sp.^37^, (**j**) *Chupacabrachelys complexus*^38^, (**k**) *Taphrosphys sulcatus*^39^, (**l**) *Notoemys laticentralis*^40^, (**m**) *Podocnemis expansa*^41^, (**n**) *Hydromedusa tectifera*^41^, (**o**) *Stupendemys souzai*^42^, (**p**) cf. *Stupendemys* sp.^43^, (**q**) and (**r**) *Stupendemys geographicus*^44^. *Ab* abduction, *Ad* adduction, *D* dorsal, *L* lateral, *M* medial, *Pr* protraction, *Re* retraction, *St* stabilization, *V* ventral. Parts (**a**–**f**) o same scale; parts (**g**–**r**) not to scale. Figure created with Adobe Creative Cloud (Adobe Inc.).

**Figure 5:**
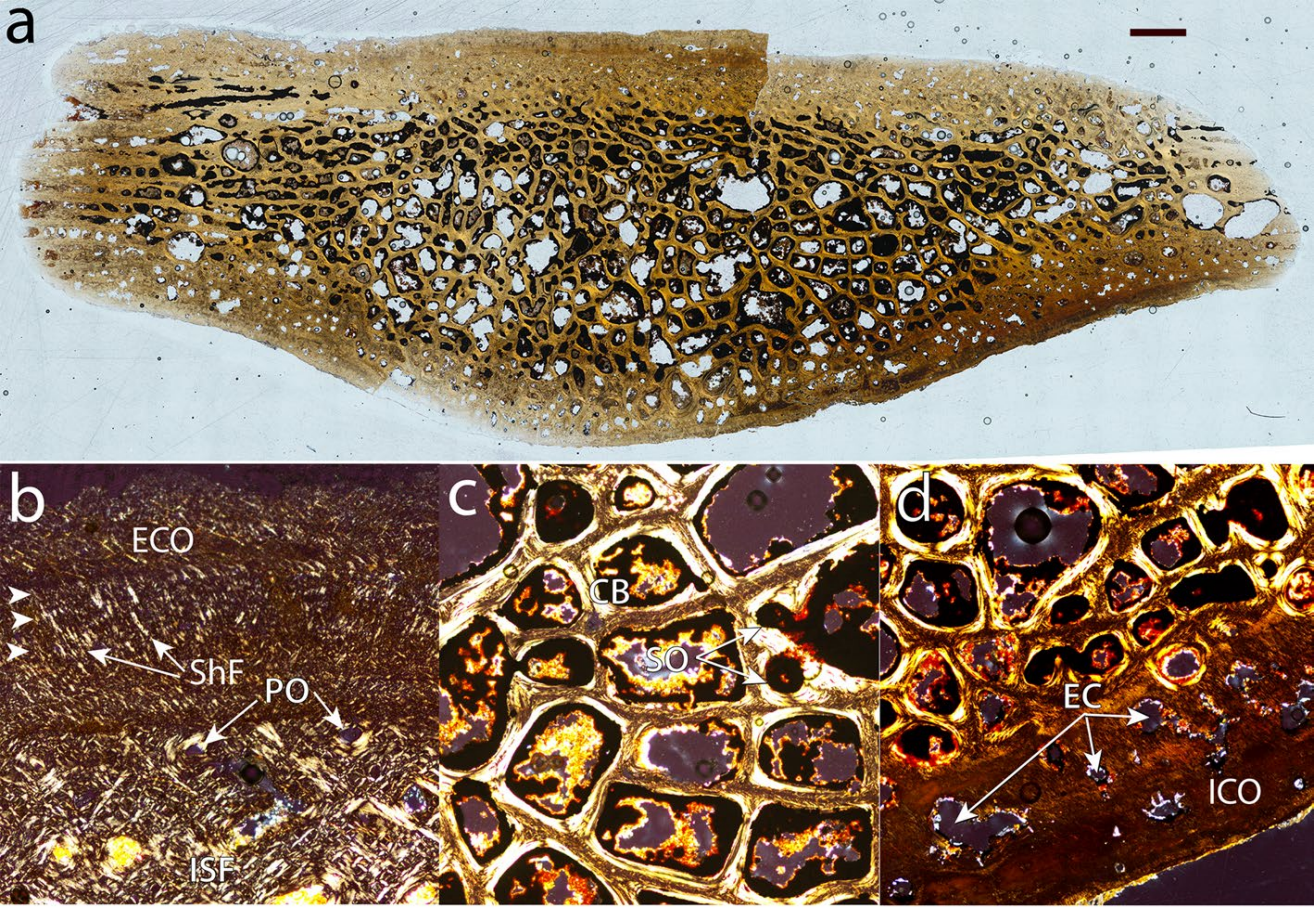
Thin section of DMNH 2013-07-0525, a probable left costal 4 of *Pleurochayah appalachius* gen. et sp. nov., from the Cenomanian (Upper Cretaceous) of the Arlington Archosaur Site (Lewisville Fm, Woodbine Gp), shown in normal transmitted (**a**) and linear-polarized light (**b**–**d**). *CB* cancellous bone, *EC* erosion cavity, *ECO* external cortex, *ICO* internal cortex, *ISF* interwoven structural collagenous fibre bundles, *PO* primary osteon, *ShF* Sharpey’s fibers, *SO* secondary osteon. Arrowheads in (**b**) indicate lamellae. Scale bar in (**a**) 1 mm; other figure parts not to scale.

*Pleurochayah appalachius* LSID: urn:lsid:zoobank.org:act:75AE0712-FB0A-436A-9069-B51AB27052C0 sp. nov. (Figs. 2, 3, 4, 5).

Type: DMNH 2013-07-1782, a partial cranium consisting of most of the basicranium, neurocranium, right otic region, and portion of the rostrum.

Type locality and horizon: Cenomanian, Upper Cretaceous. Lewisville Fm, Woodbine Gp (Fig. 1). The Arlington Archosaur Site, city of Arlington, Tarrant County, Texas. Exact locality data are on file at the Perot Museum of Nature and Science, Dallas, Texas.

Etymology: “Pleuro” is Greek for “side”, with “Cha’yah” for “turtle” in the language of the Caddo, a Native American tribe that inhabited much of the Gulf Coast of North America. Species name refers to Appalachia, the region comprising eastern North America.

Referred material: DMNH 2013-07-0683, neural 3 (cf. juvenile) (Fig. 3a–d); DMNH 2013-07-1998, pygal (Fig. 3e–h); DMNH 2013-07-0517, left costal 1 (Fig. 3i–l); DMNH 2013-07-1999, left costal 5 (Fig. 3m–p); DMNH 2013-07-1320, partial cf. right costal 3 (Fig. 3q–t); DMNH 2013-07-0557, partial cf. right costal 3 (Fig. 3u–x); DMNH 2013-07-1405, left costal 6 (Fig. 3y–ab); DMNH 2013-07-0525, partial cf. left costal 4 (Fig. 3ac–af); DMNH 2013-07-1320, partial cf. right costal 4 (Fig. 3ag–aj); DMNH 2013-07-0673, right peripheral 7 (Fig. 3ak–ap); DMNH 2013-07-1279, left peripheral 9 (Fig. 3aq–at); DMNH 2013-07-0500, proximal left humerus (Fig. 4a–g).

Diagnosis: *Pleurochayah appalachius* gen. et sp. nov. is diagnosed as a pleurodire by its quadrate with ventral process extending medially to the braincase below the cranioquadrate space, and a condylus mandibularis positioned anterior to the basioccipital-basisphenoid suture. We attribute it to Pelomedusoides by the absence of parietal-squamosal contact, and to Podocnemidoidea by a quadrate-basioccipital contact, and a prootic that is covered in ventral view. *P. appalachius* gen. et sp. nov. is a representative of Bothremydidae based on its basisphenoid-quadrate contact, a (short) quadrate-supraoccipital contact, a large exoccipital-quadrate contact, and the absence of a fossa precolumellaris. *P. appalachius* gen. et sp. nov. has the following unique combination of primitive and derived cranial characters: moderate antrum post-oticum (shared with Kurmademydini and Cearachelyini); incisura columella auris fully open and confluent with sulcus eustachii; present, but incipient, supraoccipital-quadrate contact; subtriangular, anteriorly-pointing basisphenoid; moderate posterior temporal emargination; foramen stapediotemporale located anteriorly on otic capsule, but not adjacent to foramen nervi trigemini; moderately-sized fossa pterygoidea; short parietals with uniform width; undivided fenestra postotica; external cortex of carapace significantly thicker than internal; dorsally-oriented orbits; a short quadrate-supraoccipital contact (as in Kurmademydini); distantly separate foramen stapediotemporale and foramen nervi trigemini; and lack of a slit-like fenestra postotica. See “Supplementary Information” for full anatomical description.

### Histology

See “Supplementary Information” for full histological description. The results of our histological analysis are largely consistent with other known bothremydid taxa, which have a significantly thicker external than internal cortex^45,46^. The predominance of woven bone suggests fast skeletal growth, a derived characteristic seen in *C. placidoi* and the extant pleurodiran genus *Phrynops*^46,47^. In contrast, extinct and extant cryptodires and the Cretaceous chelid *Linderochelys rinconensis* exhibit greater parallel-fibered bone, characteristic of a slower growth rate and a more conservative osteohistological pattern^46,48–53^.

### Phylogenetic placement

**Figure 6:**
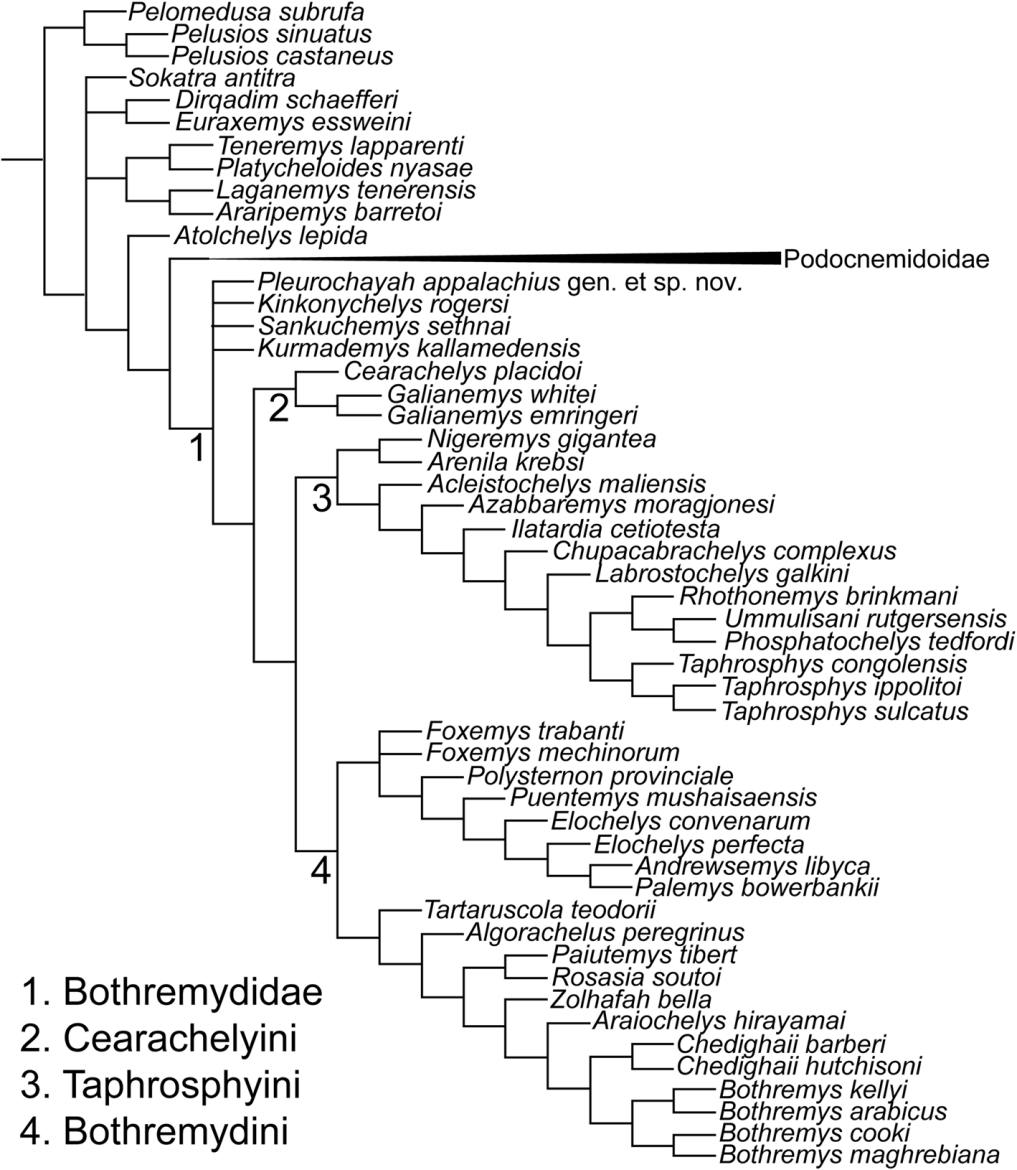
Phylogenetic position of *Pleurochayah appalachius* gen. et sp. nov. from the Arlington Archosaur Site, with strict consensus tree obtained in equal weighting. Characters are from Hermanson et al.^14^.

In the phylogenetic analysis, twenty minimum length trees of 1513 steps were obtained. In the strict consensus tree, *P. appalachius* was positioned at the base of the Bothremydidae in an unresolved polytomy with *Kinkonychelys rogersi*^54^, *Sankuchemys sethnai*^55^, and *Kurmademys kallamedensis*^56^ (Fig. 6). In 16 of the minimum trees, *P. appalachius* fell as the basal-most member of the Bothremydidae; while in the other four trees, it fell within the Kurmademydini as its basal-most member. See “Supplementary Information” for Character State Analysis and full character matrix.

## Discussion

### Comparison of the *Pleurochayah appalachius* gen. et sp. nov. cranium with other bothremydids

*Pleurochayah appalachius* gen. et sp. nov. has a unique combination of characters that differentiate it from other bothremydid taxa. Primarily, it displays many simplesiomoprhic characters that are found in Kurmademydini and Cearachelyini, but not in derived bothremydids such as Bpthremydini and taphrosphyini. Supraoccipital-quadrate contact is present but reduced compared to most other bothremydids, similar to *Kurdamemys.* The degree of posterior temporal emargination is moderate. The antrum postoticum is moderately sized, as in Kurmademydini. The foramina stapediotemporale and nervi trigemini are distant as in Kurmademydini and Cearachelyini, in contrast to the closer foramina diagnostic of Bothremydini + Taphrosphyni. The fossa precolumellaris is absent as in other early bothremydids. The quadrate process of the pterygoid is reduced compared to later bothremydids. The interorbital distance is wide, similar to that of bothremydines and *Galianemys* spp*.* The tympanic opening is elongated and oval. It lacks the slit-like fenestra postotica that characterizes cearachelyines.

*Pleurochayah appalachius* gen. et sp. nov. shares some traits with the contemporaneous *A. peregrinus*; however, most shared characters are plesiomorphic for bothremydids. The two taxa differ in many other phylogenetically significant characters. In general, the crania of both taxa are wide relative to their lengths, although this trait is common among bothremydids, and *P. appalachius* is closer in proportions to the slightly narrower *Cearachelys* than to the wider *A. peregrinus*. Prefrontal-parietal contacts are absent (as in many bothremydids). Both exhibit a short supraoccipital-quadrate contact, such that the prootic does not contact the opisthotic, as in many bothremydids. The two taxa lack a fossa precolumellaris and possess an incisura columella auris that is not completely closed, although the feature is substantially more open in *P. appalachius* gen. et sp. nov. Unlike *A. peregrinus*, the orbits in *P. appalachius* gen. et sp. nov. are more dorsally than laterally positioned. A lateral, more vertical orientation of the orbits is considered primitive among turtles, and the most common state for bothremydines^10^. However, dorsal and more horizontally oriented orbits are a synapomorphy for *Bothremys* spp. and *Chedighaii* sp.^10^. There is not a clear functional distinction between the lateral and dorsal positions of the orbits, though in chelonioids it has been hypothesized that dorsal orientation is associated with shallow-water and/or benthic habits, and vertical orientation with pelagic adaptation^57^.

The fossa orbitalis is posteriorly enlarged in *A. peregrinus*, but not in *P. appalachius* gen. et sp. nov., in which the septum orbitale is only slightly posterior to the orbital rim. There is extensive contact between the postorbital and frontal in *A. peregrinus*, but only a minimal point of contact in *P. appalachius* gen. et sp. nov. The foramen stapediotemporale is located further from the foramen nervi trigemini in *P. appalachius* gen. et sp. nov., although in both taxa the foramen stapediotemporale is positioned anteriorly on the otic chamber. *Pleurochayah appalachius* gen. et sp. nov. has a short crista supraoccipitalis, as opposed to the long crista supraoccipitalis in *A. peregrinus.*

**Figure 7:**
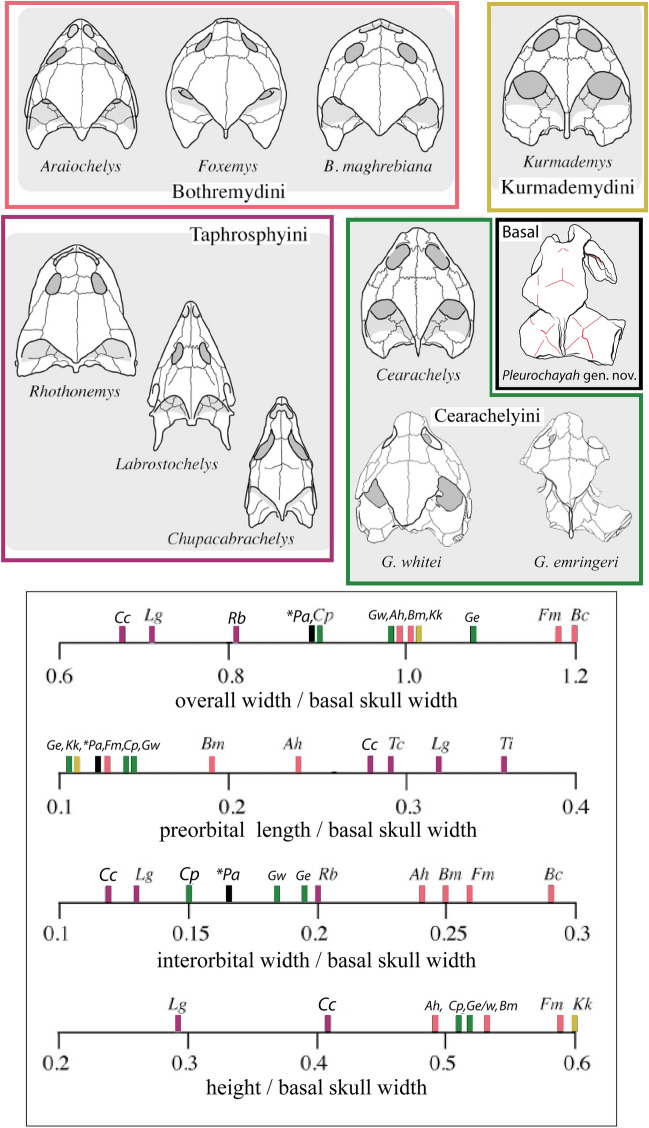
Cranial comparison of selected Bothremydidae skulls in dorsal view, standardized to comparable length. Scales show three ratios of standard skull measurements for bothremydids: (***Ah***) *Araiochelys hirayamai*, (***Bc***) *Bothremys cooki*, (***Bm***) *Bothremys maghrebiana*, (***Cc***) *Chupacabrachelys complexus*, (***Cp***) *Cearachelys placidoi*, (***Fm***) *Foxemys mechinorum*, (***Ge***) *Galianemys emringeri*, (***Gw***) *Galianemys whitei*, (***Kk***) *Kurmademys kallamedensis*, (***Lg***) *Labrostochelys galkini*, (*Pa) *Pleurochayah appalachius*, gen. et sp. nov.*,* (***Rb***) *Rhothonemys brinkmani*, (***Tc***) *Taphrosphys congolensis*, (***Ti***) *Taphrosphys ippolitoi*. Modified from Lehman and Wick^38^, with skull reconstructions of *G. whitei* and *G. emringeri* from Gaffney et al.^58^, and remaining skull reconstructions and ratios based on measurements from Gaffney et al.^10^. Parts of figure not to scale. Figure created with Adobe Creative Cloud (Adobe Inc.).

Using ratios of basal skull width to other measures, Fig. 7 compares general skull proportions of a selection of bothremydid taxa. The ratio of the overall width of the skull to basal skull width in *P. appalachius* gen. et sp. nov. is 0.87, similar to the cearachelyine *C. placidoi*, which falls between the narrow Taphrosphyini (< 0.82) and broader Bothremydini and *K. kallamedensis* (> 0.97) (Fig. 7). The preorbital length to basal skull width ratio of *P. appalachius* gen. et sp. nov. is also quite low, embedded in a cluster with cearachelyines, *K. kallamedensis*, and *F. mechinorum* (Fig. 7). In the ratio of interorbital width to basal skull width, *P. appalachius* gen. et sp. nov. falls within the known taphrosphyine and cearachelyine ranges (Fig. 7). A similar intermediate position within the bothremydine spectrum of variation, is repeated in the height to basal skull width ratio (Fig. 7). However, *P. appalachius* gen. et sp. nov. could not be included in the final comparison due to its height being diagenetically altered by crushing. In summary, compared to other bothremydids known from skulls, *P. appalachius* gen. et sp. nov. has a moderately narrow skull with a very short face and moderately spaced orbits (Fig. 7).

### Humeral comparison between *Pleurochayah appalachius* gen. et sp. nov. and other pleurodirans

Previous studies used humeral morphology to infer locomotor modes and performance^41,59–62^ and paleoecology^63^ in turtles. Figure 4 provides a comparison of all published fossil pleurodiran humeri, along with a few extant taxa. The proximal humerus of pleurodires generally has a hemispherical head that is slightly separated from the lateral and medial processes, but varies in proportional length and width, and in orientation relative to the humeral shaft^10^ (Fig. 4). A more spherical humeral head is considered the derived state in bothremydids^10^ (Fig. 4). The relative proportions of the humeral head (i.e., length to width) are nearly equal in *P. appalachius* gen. et sp. nov., similar to most specimens of *Stupendemys* spp.*, Taphrosphys sulcatus,* and *Hydromedusa tectifera* (Fig. 4). In contrast, in *Bothremys barberi*, *Bothremys* sp., *Chupacabrachelys complexus*, and the extant *Podocnemis expansa*, the humeral head is slightly longer than wide, while in *Notoemys laticentralis*, the humeral head is wider than long (Fig. 4). The longitudinal axes of the humeral heads of *P. appalachius* gen. et sp. nov. and *Taphrosphys sulcatus* are parallel with the humeral shaft (Fig. 4). However, in *Bothremys barberi*, *Bothremys* sp., *Chupacabrachelys complexus*, *Notoemys laticentralis*, *Podocnemis expansa*, *Hydromedusa tectifera*, and *Stupendemys* spp., the longitudinal axis is tilted slightly laterally and the difference between the heights of the processes is generally greater, echoing this tilt (Fig. 4).

The lateral and medial humeral processes are homologous with the trochanter major and minor, respectively^61^. Usually, the medial process is taller and more robust than the lateral, and the difference in height varies significantly between taxa (Fig. 4). The medial process is only slightly taller than the lateral process in *P. appalachius* gen. et sp. nov., similar to *Taphrosphys sulcatus*, but the difference in heights is more notable in the remainder of the comparative sample (Fig. 4). The lateral process is shifted significantly distally in sea turtles and to a lesser degree in *Carettochelys insculpta*^34^. The humeral head of *P. appalachius* gen. et sp. nov. is taller than both processes, as in all compared taxa except the chelid *Hydromedusa tectifera*, *Notoemys laticentralis*, and most humeri of *Stupendemys* spp. (Fig. 4). The neck of the medial process is slightly depressed, forming a sloping saddle in all compared taxa except *Notoemys laticentralis* (Fig. 4).

The large proximal processes of semiaquatic turtle humeri increase the mechanical advantage of both the m. pectoralis major and m. latissimus dorsi supporting more powerful stroke and recovery during both aquatic rowing and terrestrial walking^64–66^. Muscles inserting on the proximal aspects of the lateral and medial processes act primarily to adduct and retract the humerus, while the more distal insertions are associated with protraction and abduction. In general, the lateral process tends to be associated with anterior movement of the limb, and the medial with posterior movement^34^. The lateral process is also relatively proximal compared to other sampled turtles, and is most similar to the marine-adapted *Taphrosphys sulcatus* (Fig. 4). Lateral and medial processes of similar height and size, as in *P. appalachius* gen. et sp. nov., suggests that adduction and retraction of the forelimb may be comparably distributed between the anterior and posterior directions, resulting in more equally powerful stroke and recovery motions during rowing. This is in contrast to an emphasis on posterior forelimb movement such as the flapping motion of many marine turtles usually associated with an enlarged medial process^34^.

The proximal humeral morphology of *P. appalachius* gen. et sp. nov., including a derived spherical head and robust medial and lateral processes similar to marine-adapted *Taphrosphys sulcatus,* suggests well-developed near-marine aquatic capabilities. Recovery of more skeletal material is needed to further test this intriguing hypothesis. While it should be noted that the described postcranial material was not found in direct association with the *P. appalachius* DMNH 2013-07-1782 cranium, we believe it to be the most parsimonious explanation that they belong to the same taxon, rather than the alternative that two previously undescribed pleurodiran species were present at AAS.

### Histological comparison of *Pleurochayah appalachius* gen. et sp. nov. with fossil and extant pleurodires

In his seminal work on turtle shell histology, Scheyer^45^ developed a system of categories to quantify and relate histological characteristics and trends to the degree of aquatic adaptation of fossil and recent turtles. These categories are based on published ecological data of recent turtles^67–70^, and though a strict adherence to categorization should be treated with caution, a consideration of *P. appalachius* gen. et sp. nov. in the context of the analysis of Scheyer^45^ yields possible paleoecological insights into the new taxon.

**Figure 8:**
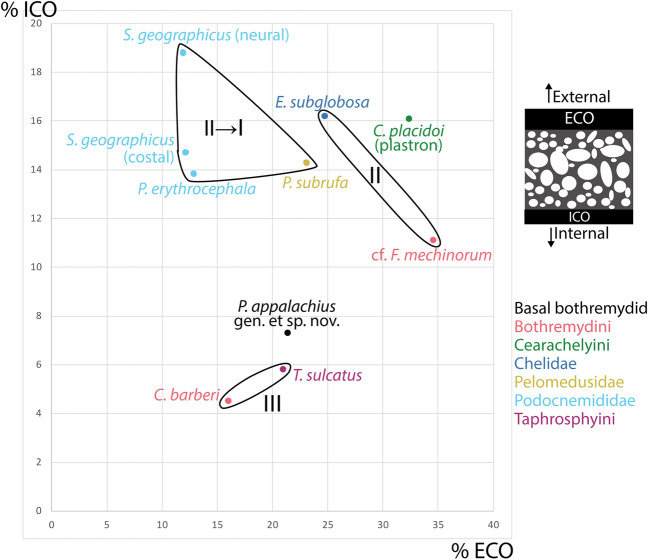
Results of histological comparison of fossil and extant pleurodiran taxa, comparing relative thicknesses of internal (**ICO**) and external cortices (**ECO**) to total shell thickness. Roman numerals indicate ecological adaptation categories of Scheyer^45^: *I* terrestrial, *II* semiaquatic to mainly aquatic, and *III* fully aquatic. Measurements taken from Scheyer and Sánchez-Villagra^72^. Assignment to categories from Scheyer^45^, Appendix 3. Schematic at upper right shows generalized cross section of diploë structure in turtle shell.

Figure 8 compares the percentage thickness of the external and internal cortices relative to total shell bone thickness. *P. appalachius* gen. et sp. nov. falls closest to the marine-adapted bothremydids *Chedighaii barberi* and *Taphrosphys sulcatus* (Fig. 8). Comparative turtle taxa included here fall within Category II (semiaquatic to mainly aquatic) and Category III (fully aquatic) in degree of aquatic adaptation^45^. Categories II and III were combined into the Freshwater Histological Type of Jannello et al*.*^71^, which includes marine coastal environments. Turtles in Category II spend much of their lives in the water, often going onto land to migrate, forage, and bask^45^. They all have shell bones with a diploë microstructure, in which internal and external cortical bone layers frame interior cancellous bone^45^ (Fig. 8). The cortices are well developed, and the histological profile of the bone has a generally compact appearance^45^. Vascularization of the cortical bone is higher than terrestrial (Category I) turtles due to more primary vascular canals and secondary osteons^45^. Also, the transitions between the cortices and interior cancellous bone remain conspicuous^45^. Among the currently sampled taxa, the bothremydid *Foxemys* cf. *mechinorum* and chelid *Emydura subglobosa* belong to Category II, as do the sampled Podocnemididae (*Stupendemys geographicus* and *Podocnemis erythrocephalica*) and the pelomedusid *Pelomedusa subrufa*, though the latter also has tendencies toward Category I (Fig. 8). Turtles in Category III (the bothremydids *Chedighaii barberi* and *Taphrosphys sulcatus)* are characterized by: rarely leaving an aquatic environment to bask or lay eggs; reduction in compact bone layers in the shell; strong reduction in thickness of the internal cortex compared with the external cortex; strongly vascularized external cortex that is rarely reduced in thickness; and highly organized trabeculae in the cancellous bone^45^. A well-developed external cortex is hypothesized to ensure structural stability of the shell bones^45^. The clustering of *P. appalachius* gen. et sp. nov. with the Category III bothremydids (Fig. 8) is intriguing and suggests a highly aquatic, possibly semi-marine lifestyle. This functional interpretation is consistent with the morphology of the humerus.

Unlike the consistent account of aquatic adaptation shared by the sampled Podocnemididae, the bothremydid *Foxemys* cf. *mechinorum* shows less aquatic adaptation in its microanatomy than the marine bothremydid taxa *Bothremys barberi* and *Taphrosphys sulcatus*^45^ (Fig. 8)*.* Also, *P. appalachius* gen. et sp. nov. has similar cortex to full shell thickness ratios as the marine taxa, further suggesting that it may also have been highly adapted to aquatic life (Fig. 8). Additional discoveries of shell and postcranial material may reveal further paleoecological insights^63,73^ on *P. appalachius* gen. et sp. nov. and related taxa. In particular, histological sampling of *Algorachelus* spp. to measure relative cortical thickness and other relevant characteristics may be useful in assessing the paleoecology of these taxa.

### Pleurodiran distribution and southern Appalachian biogeography

As Kurmademydini is not retrieved in the current phylogenetic analysis, *Pleurochayah appalachius* gen. et sp. nov. is part of an unresolved basal bothremydid polytomy along with *Sankuchemys sethnai*^55^, *Kinkonychelys rogersi*^54^, and *Kurmademys kallamedensis*^56^ (Fig. 6). Basal bothremydids are known from Africa, South America, India, and now North America. The group originated in Gondwana during the early Cretaceous and secondarily colonized Europe and North America in the late Cretaceous^13^. The analysis of Joyce et al.^13^ implies that at least five separate clades invaded North America from Gondwana. In addition to the new taxon described herein, these migrations include the newly established ancestors of *Paiutemys tibert* prior to the Cenomanian, the previously established ancestors of *Chedighaii* and newly established ancestor of *Chupacabrachelys complexus* prior to the Campanian, the previously established ancestors of *Bothremys cooki* prior to the Maastrichtian, and the newly established ancestor of *Taphrosphys sulcatus* prior to the Paleocene^13^.

The vast majority of bothremydines are preserved in near-shore marine deposits surrounding the Atlantic Ocean^10^, while basal bothremydids are recovered from continental sediments^2,10,13^. Trans-Tethyan dispersal events (from northern Gondwana to the European archipelago) appear to be responsible for the movement of many faunal groups during the early Upper Cretaceous, particularly during the Cenomanian–Santonian interval^74^. The southwest coast of Appalachia has a similarly complex biogeographical system and lies along a land connection and migration route from Laramidia^75^. The AAS also occupies a unique geographical location near the opening of the Western Interior Seaway, which connected the Arctic to the Gulf of Mexico beginning in the late Albian^31^. As such, it was situated near a confluence of brackish and near-shore marine waters with continental drainages from the Ouachita Mountains to the north^17^. Marine adaptation was previously known as early as the Santonian among the Bothremydini, as *Bothremys arabicus* and *Chedighaii* sp. or *Bothremys barberi* likely had near-shore marine lifestyles^76^. Freshwater pleurodiran taxa experienced reduced diversity in the Albian to Campanian interval, while the littoral and marine taxa showed a trend of steadily increasing diversity over the same period^77^. The shell histology of *P. appalachius* gen. et sp. nov. provides an example of a basal bothremydid with comparable marine adaptation to bothremydines (Fig. 8), occurring near the beginning of the trend of increased marine diversity.

A partial adaptation of some bothremydid subclades to brackish and marine waters facilitated their dispersal and the invasion of different niches. More than vicariance or large-scale extinctions, dispersal events are considered the most important influence on biogeography in pleurodires^77^. Oceanic dispersals are known to be a significant cause of biogeographic range changes for other groups such as tortoises^78,79^, lizards^80^, amphibians^81^, and invertebrates^82^. Some pelomedusids and chelids have island distributions that suggest short dispersals^77^, and extant freshwater turtles *Chelodina expansa* and *Emydura macquarii* have been exposed to saline conditions for long periods (50 days) without physiological problems^83^. Consistent with this pattern of increased marine adapation in the mid-Cretaceous, *Pleurochayah appalachius* gen. et sp. nov. demonstrates gross morphological and paleohistological signatures of marine or highly aquatic brackish adaptations. This suggests that marine adaptations among stem-pleurodires were earlier and more widespread than previously thought^77^. In the context of the AAS paleoenvironment, *P. appalachius* gen. et sp. nov. joins a number of previously described brackish or marine-tolerant taxa including the crocodyliform *Terminonaris*, hybodont sharks, and multiple invertrebrates that are consistent with a costal setting where terrestrial, freshwater, and marine taxa intermingle^20,84,85^.

## Conclusions

**Figure 9:**
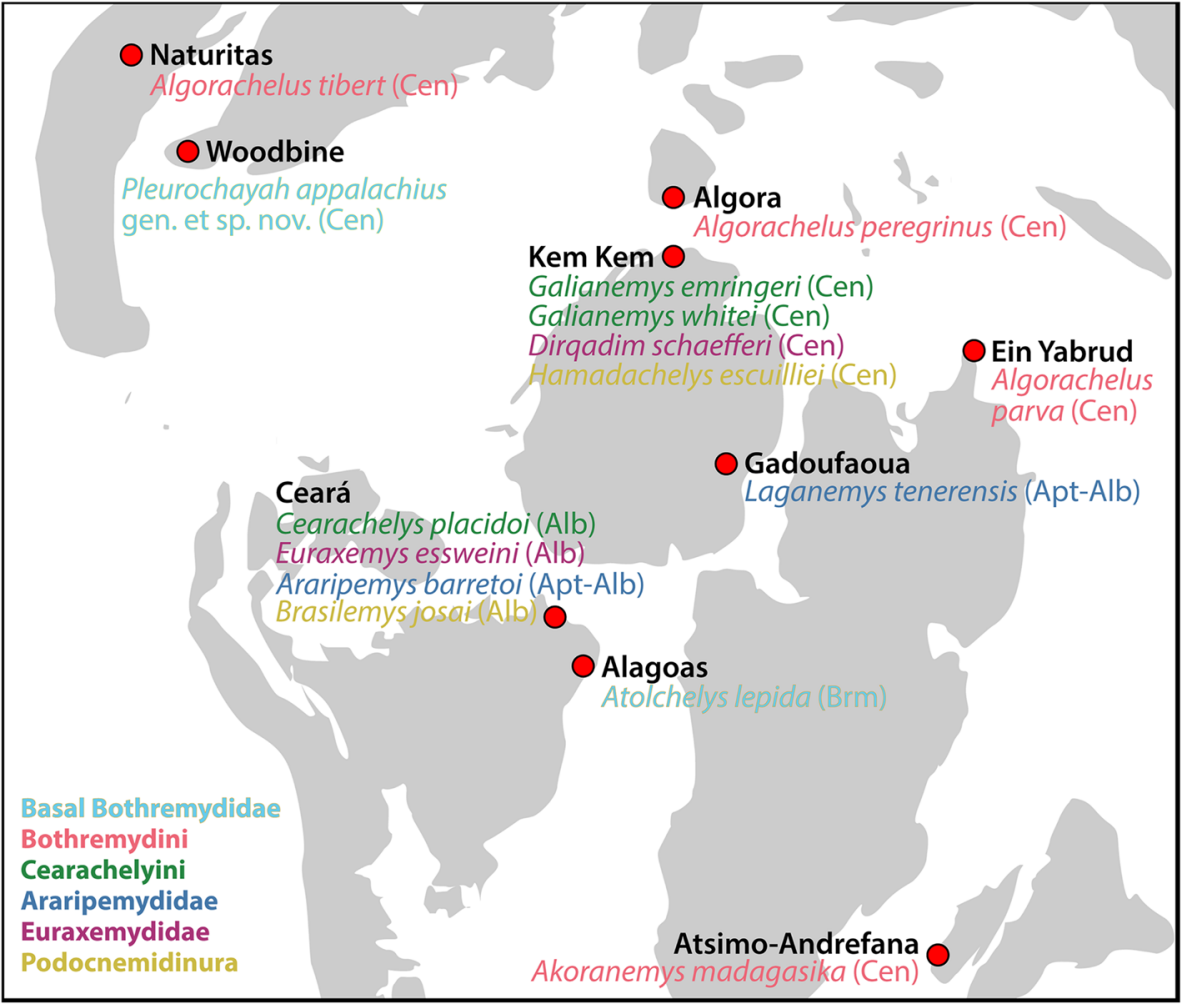
Geographical position of the type localities (red circles) of known fossil crown pleurodires in Laurasia and northern Gondwana during and prior to the Cenomanian (Upper Cretaceous). Modified from global Mollweide projection at 96.6 Ma by Scotese^87^. Colors of taxon text corresponds with clades at bottom left. *Alb* Albian, *Apt–Alb* Aptian/Albian, *Brm* Barremian, *Cen* Cenomanian. Figure created with Adobe Creative Cloud (Adobe Inc.).

The current study places *Pleurochayah appalachius* gen. et sp. nov. at the base of the Bothremydidae, in an unresolved polytomy with the members of Kurmademydini (Fig. 6). It is the oldest known crown pleurodire and bothremydid from North America. This study also extends the geographic range of basal bothremydids to North America, which is significant since they were previously known only from South America prior to the Cenomanian (Fig. 9). *Pleurochayah applachius* gen. et sp. nov. is known from the lower middle Cenomanian, predating *Algorachelus peregrinus* in the uppermost middle-lowermost upper Cenomanian^9^, and *Pauitemys tibert* in the uppermost Cenomanian^13^, making it the oldest known pleurodire from North America and Laurasia. It is likely that bothremydid dispersals to North America during or prior to the Cenomanian were influenced by the vicariant event of the opening of the central Atlantic, and the periodic connection of the Western Interior Seaway to the Gulf of Mexico. However, allopatric speciation may be responsible for the multiple-continent distribution of basal bothremydids, as demonstrated for other late Early Cretaceous pelomedusoid clades. Recent discoveries of new bothremydid taxa in Central America^86^ suggest additional insight into circum-Carribbean pleurodiran migrations may be possible.

## Materials and methods

Fossil specimens were collected and prepared by researchers, staff, and volunteers of the Perot Museum of Nature and Science. There were no definitive associations between the cranium and postcranial material. Specimens were measured with 6″ Mitutoyo Absolute Digimatic calipers to the nearest 0.01 mm and rounded to the nearest 0.1 mm. Some distances and angles were measured from high quality digital photographs using ImageJ^88^.

For our phylogenetic analysis of *P. appalachius* gen. et sp. nov., we used the recent character matrix of 285 characters from Hermanson et al.^14^ designed to reveal phylogenetic relationships within Pleurodira. Characters following a morphocline were ordered according to Hermanson et al.^14^. Phylogenetic analyses were performed in TNT 1.5^89^. We conducted a traditional search using 1000 replicates of Wagner trees and tree bisection-reconnection (TBR) saving 100 trees per replication. The full character matrix derived from Hermanson et al.^14^ is available in “Supplementary Information”.

We apply the taxonomic scheme of turtles presented by Joyce^90^ unless otherwise specified. Phylogenetically defined clade names are used in accordance with PhyloCode guidelines^91,92^.

For histological thin-sectioning, DMNH 2013-07-0525 was left undecalcified and embedded in plastic resin following the protocol of Lee and Simons^93^. Slides were imaged using a motorized light microscope (Ni-U; Nikon, Tokyo, Japan, USA) with a strain-free long working distance objective (10× Plan Fluor: numerical aperture of 0.3, resolvable size ≈ 1 μm). Focus and stitching of histological montages were controlled by software (NIS Elements D; Nikon, Tokyo, Japan, USA). The montages were sharpened using Photoshop (CC; Adobe, San Jose, California, USA) with the “Unsharp Mask” filter set at 10 px and are high resolution (2.1 μm per pixel). To comply with major grant funding agencies and promote data transparency, we provide the images as freely accessible digital slides at the Paleohistology Repository (Lee and O’Connor^94^: http://paleohistology.appspot.com).

## Supplementary Information

Supplementary Information 1.

Supplementary Information 2.
