# Supplementary material for: An early bothremydid from the Arlington Archosaur Site of Texas
Source: Sci Rep. 2021 May 20;11:9555. doi: 10.1038/s41598-021-88905-1 (PMC8137945; doi:10.1038/s41598-021-88905-1)
Supplement: Supplementary file 1 — Supplementary Information 1. [file 41598_2021_88905_MOESM1_ESM.docx]

# An early bothremydid from the Arlington Archosaur Site of Texas

# Brent Adrian, Heather F. Smith, Christopher R. Noto, and Aryeh Grossman

**SUPPLEMENTARY INFORMATION**

1. Expanded Geological Background

2. Abbreviations

3. Anatomical Description

4. Histological Description

5. Character State Analysis

6. Additional References

7. Table

**1. Expanded Geological Background**

The Arlington Archosaur Site (AAS) preserves the terrestrial facies of the Woodbine Group, the only terrestrial rocks known from the Cretaceous of Central Texas after the mid-Albian (Winkler *et al.*, 1995) (Fig. 1). The AAS deposits include, in ascending order: a carbonaceous sandy siltstone (peat bed) representing a freshwater or brackish coastal wetland (facies A); a gray siltstone paleosol (vertisol or gleysol) with a heavily rooted zone containing calcareous concretions associated with burned logs and tree stumps, indicating seasonal dryness and wildfires (facies B); siderite-cemented silt nodules and a sand transgressive lag (facies C); and alternating layers of gray siltstone and fine sand, with some preserving ripples marks, representing a marine-influenced environment of cyclic deposition such as an intertidal mudflat (facies D) (Bennett *et al.*, 2011; Noto, 2015; Adams *et al.*, 2017). Facies A comprises the primary and most productive fossil quarry at the AAS, and has yielded the specimens presented here and in previous studies (Adams *et al.*, 2011, 2017; Noto *et al.*, 2012, 2019; Adrian *et al.*, 2019). The AAS has produced a particularly diverse crocodyliform fauna, including teeth and skeletal remains belonging to *Woodbinesuchus*, *Terminonaris*, and new taxa *Deltasuchus motherali* and *Scolomastax* *sahlsteini* (Adams *et al.*, 2017; Noto *et al.*, 2019). Dinosaurs include skeletal remains and teeth attributed to the basal hadrosauroid *Protohadros*, as well as teeth and fragmentary postcranial remains from allosauroid, tyrannosauroid, and dromaeosaurid theropods (Main *et al.*, 2014; Noto, 2015, 2016). The fossil-bearing exposures are organically rich and contain abundant arenaceous foraminifers, molluscs, and ammonites (Kennedy and Cobban, 1990; Trudel, 1994; Noto, 2015; Adrian *et al.*, 2019). Strata are dominated by blue-gray to black, finely laminated to massive-bedded mud and shale interspersed with glauconitic sandstone lenses (Oliver, 1971; Trudel, 1994; Main, 2005; Noto, 2015).

Environmental reconstructions of the site show a coastal environment, with geology and fossils that reflect a mixture of terrestrial, freshwater, and marine influences that increase over time (Adams *et al.*, 2017; Main, 2013; Noto *et al.*, 2012, 2019). The AAS preserves a diverse coastal community that inhabited a low delta plain created during a regression of the southeastern margin of the Western Interior Seaway (WIS) (Stephenson and Stenzel, 1952; Oliver, 1971; Main, 2005; Adams and Carr, 2010) (Fig. 1c). This community includes plants (MacNeal, 1958; Dilcher and Crane, 1984), invertebrates (Stephenson and Stenzel, 1952), dinosaur bones, teeth and tracks (Lee, 1997a, b), as well as crocodyliforms (Adams *et al.*, 2011, 2017; Noto *et al.*, 2019), turtles (Main, 2005; Noto *et al.*, 2012; Main *et al.*, 2013; Adrian *et al.*, 2019), and mammals (Krause and Baird, 1979). Surface collecting and screen washing have revealed a diverse ichthyofauna, including chondrichthyans (an indeterminate hybodont, *Cretodus*, *Pseudohypolophus*, and *Onchopristis*) and osteichthyans (an indeterminate pycnodont, lepisosteiforms, cf. *Stephanodus*, and dipnoan *Ceratodus carteri*) (Bennett *et al.*, 2011; Main *et al.*, 2011; Main, 2013; Noto, 2015). At least four turtle taxa are known from the AAS, including the helochelydrid *Naomichelys* sp., the baenid “*Trinitichelys” maini*, an indeterminate trionychid, and the pleurodire described here (Adrian *et al.*, 2019). Adrian *et al.* (2019) assigned a single costal to *Algorachelus* sp., a genus known from Cenomanian rocks in the Iberian Peninsula and Palestine (Joyce *et al.*, 2016; Pérez-García, 2018). We re-assess this specimen in light of the new material available.

**2. Abbreviations**

**Anatomical abbreviations — bo**= basioccipital, **bs**= basisphenoid, **CB**= cancellous bone, **cm**= condylus mandibularis, **co**= condylus occipitalis, **cs**= crista supraoccipitalis, **ECO**= external cortex, **ex**= exoccipital, **fjp**= foramen jugulare posterius, **fnt**= foramen nervi trigeminale, **fp**= fossa pterygoidea, **fpcci**= foramen posterius canalis caroticus internus, **fr**= frontal, **fst**= foramen stapediotemporale, **ica**= incisura columella auris, **ICO**= internal cortex, **ISF**= interwoven structural collagenous fibre bundles, **Ma**= marginal scale, **mx**= maxilla, **op**= opisthotic, **or**= orbit, **pa**= parietal, **pal**= palatine, **pf**= prefrontal, **Pl**= pleural scale, **pm**= premaxilla, **po**= postorbital, **PO**= primary osteon, **pr**= prootic, **pt**= pterygoid, **qu**= quadrate, **ShF**= Sharpey’s fibres, **so**= supraoccipital, **SO**= secondary osteon, **sq**= squamosal, **tb**= tuberculum basioccipitale, **to**= tympanic opening, **Ve**= vertebral scale, **xi**= xiphiplastron, **XII**= nervi hypoglossi.

**Institutional abbreviations** —

**AMNH** American Museum of Natural History, New York, New York, USA

**BSP** Bayerische Staatssammlung für Paläeontologie und Historische-

Geologie, Munich, Germany

**DMNH** Perot Museum of Nature and Science (formerly the Dallas Museum of Paleontology), Dallas, Texas, USA

**FM** The Field Museum, Chicago, Illinois, USA

**IPB** Goldfuss-Museum, Institute for Paleontology, University of Bonn, Bonn, Germany

**MN** Museu Nacional, Universidade Federal do Rio de Janeiro, Rio de Janeiro, Brazil

**MPSC** Museu Paleontológico de Santana do Cariri, Santana do Carriri, Brazil

**MVZ** Museum of Zoology, University of California, Berkeley, California, USA

**THUg** Teikyo Heisei University, Chiba, Japan

**UNEFM-CIAPP** Centre of Archaeology, Anthropology and Paleontology of Francisco de Miranda University, Coro, Venezuela

**YPM** Yale Peabody Museum, New Haven, Connecticut, USA

**3. Anatomical Description**

**Skull**

The holotype of *P. appalachius* gen. et sp. nov. is a partial cranium, DMNH 2013-07-1782, consisting of most of the basicranium, cranial vault, right otic region, and part of the right rostrum (Fig. 2). The neurocranium preserves much of both parietals, both frontals, part of the prefrontals, and part of the left postorbital (Fig. 2a-b). The basicranium includes the basioccipital, basisphenoid, exoccipitals, and portions of both pterygoids, which are slightly distorted (Fig. 2e-f). The otic regions preserve the opisthotics, supraoccipitals, and prootics (Fig. 2). Portions of the quadrate are present on both sides, but are more complete on the right side (Fig. 2). The rostral region is represented primarily by the right maxilla and premaxilla (Fig. 2a-b, e-h). The absent temporal arches, jugals, and incomplete maxillae permit a view of the interorbital region (Fig. 2c-d, g-h).

*Dermal roofing elements.* Only small portions of the prefrontals are preserved in DMNH 2013-07-1782 (Fig. 2a-f), and they form a portion of the dorsal margin of the crushed apertura narium externa. They are convex dorsally in contrast to the flattened prefrontal in some pelomedusids (Gaffney *et al.*, 2001). Ventrally, the prefrontal forms the anterior margin of the foramen interorbitale. A small portion of the left postorbital is present (Fig. 2a-f). It contacts the parietal posterodorsally, and frontal anterodorsally along the dorsal orbital margin (Fig. 2a-d). The frontals are roughly rectangular, and contact the parietals posteriorly, prefrontals anteriorly, and each other at the midline (Fig. 2a-d). The frontal is connected to the postorbital by a small sliver of bone along the dorsal orbital margin (Fig. 2a-b). The frontals comprise the majority of the dorsal margins of the orbits (Fig. 2a-d). A sufficient portion of the orbit is present in *P. appalachius* gen. et sp. nov. to conclude that its orbits are widely spaced, rounded, and face more dorsally than laterally, but not as dorsally positioned as *Bothremys* or *Chedighaii* (Gaffney *et al.*, 2006). On the right side, the interorbital region is visible, including the vertical portion of the frontal (Fig. 2g-h). There is a large foramen interorbitale along the ventral margin of the frontal, which does not extend posteriorly to the parietal.

The paired parietals are roughly rectangular in shape (Fig. 2a-d, g-h), and do not widen posteriorly as in most bothremydids, but remain uniformly wide as in *C. placidoi* (Gaffney *et al.*, 2001). They contribute to a moderate degree of temporal emargination, dorsally exposing the otic region almost entirely, and are separated from the posterodorsal margin of the orbits (Fig. 2a-b). The processus inferior parietalis forms the lateral wall of the braincase, along with the pterygoid and prootic (Fig. 2c-d). It forms the anterodorsal margin of the foramen nervi trigemini, in conjunction with the pterygoid and prootic. The squamosals are missing bilaterally, but the small quadrate-squamosal suture on the right side suggests a relatively small squamosal bone with no contribution to the tympanic opening (Fig. 2g-h). The postorbital forms the posterodorsal margin of the orbit, and contributes to the moderate temporal emargination (Fig, 2a-d).

*Palatal elements.* The premaxilla is roughly triangular in shape, tapering posteriorly (Fig. 2a-b, g-h). There is a distinct but thin rounded labial ridge anteriorly (Gaffney *et al.*, 2001, 2002). Posterior to the ridge, a thin groove runs parallel to the external margin of the bone, and then the bone becomes slightly convex. Posteromedially, the premaxilla bounds a narrow, ovoid apertura narium interna (Fig. 2e-f). Despite the dramatic compression of the fossa nasalis in DMNH 2013-07-1782, it is still clear that the premaxilla contributed to its anterior floor and the ventral margin of apertura narium externa.

Most of the right maxilla of DMNH 2013-07-1782 is preserved (Fig. 2a-b, e-h), articulating with the premaxilla and palatine. The medial aspect of the horizontal plate forms the lateral margin of the deformed apertura narium externa. The triturating surface is smooth and lacks the deep pits found in Bothremydini (Gaffney *et al.*, 2006) (Fig. 2e-f). It consists of a narrow, sharper labial ridge and broader lingual ridge, separated by a V-shaped trough. The maxilla expands slightly laterally to form a convex outline (Gaffney *et al.*, 2006) (Fig. 2a-b, e-f). The maxilla comprises the ventral margin of the orbit, which is large and rounded, despite being incomplete in this specimen (Fig. 2e-f). The orbit is more dorsally than laterally positioned, but lacks the extremely dorsal position of some Bothremydini (Gaffney *et al.*, 2006). The horizontal plate forms the lateral portion of the floor of the orbit. The lateral palatal margins are missing bilaterally, so it is not possible to assess the extent of the palatine contribution to the triturating surface (Fig. 2e-f). The palatine-pterygoid suture is obscured and partially absent, and therefore the foramen palatinum posterius is not preserved.

*Palatoquadrate elements.* The ventral process of the quadrate extends medially to the braincase below the cranioquadrate space, contacting the basisphenoid and basioccipital (Fig. 2e-f). The foramen posterior canalis caroticus inferior is in the basisphenoid-pterygoid suture, but approximately at the contact of the quadrate, basisphenoid, and pterygoid (Fig. 2e-f). The foramen stapediotemporale is positioned anteriorly on the otic chamber in the quadrate-prootic suture (Fig. 2g-h). The cavum tympani is comprised entirely of the quadrate with minimal apparent contribution from the quadratojugal or squamosal (Fig. 2g-h). It is ovoid, and its most prominent feature is an open incisura columella auris (Fig. 2g-j). The incisura is rounded as in *C. placidoi*, as opposed to the slit-like condition of *Foxemys* spp. (Gaffney *et al.*, 2006). There is no fossa precolumellaris, as with many Bothremydidae. The antrum postoticum is small, although may be eroded laterally. Laterally, the quadrate forms the roof of the incisura columella auris and fenestra postotica. There is no apparent separation between the foramina for the vena capitis lateralis and stapedial artery. The condylus mandibularis is narrow and flattened ventrally and positioned anterior to the condylus occipitale at approximately the level of the basioccipital-basisphenoid suture (Fig. 2e-j).

The pterygoids are roughly rectangular (Fig. 2e-f) and separated posteriorly by the basisphenoid. A broken lateral projection may represent the remainder of a processus trochlea pterygoidei. The pterygoid forms the lateral margin of a moderately sized fossa pterygoidea (Gaffney *et al.*, 2001, 2002). The quadrate process does not extend posterior to the basisphenoid/pterygoid suture (Fig. 2e-f) (Gaffney *et al.*, 2001). At the midline, the suture with the palatines is straight, but the lateral extent of the suture is not present.

*Braincase elements*. The supraoccipital-quadrate connection separates the prootic and opisthotic, as in other bothremydids (Fig. 2a-b). However, the supraoccipital-quadrate contact is extremely reduced compared to most bothremydids (Gaffney *et al.*, 2006). The supraoccipital forms the dorsal and dorsolateral margins of the foramen magnum (Fig. 2i-j). The crista supraoccipitalis is short and narrow and does not project posteriorly beyond the margin of the foramen magnum (Fig. 2a-d). There is a small depression at approximately the position of the supraoccipital-prootic suture, which superficially resembles a foramen stapediotemporale; however, close inspection revealed that this feature is a taphonomic depression rather than a biological feature (Fig. 2a-b). The posterior margin of the cranium, surrounding the ventral margin of the foramen magnum and condylus occipital, is partially crushed (Fig. 2e-f, i-j).

The exoccipitals do not meet at the midline dorsal to the foramen magnum (Fig. 2i-j). While it is apparent that the exoccipitals form the ventrolateral margins of the foramen magnum, the extent of their ventral contribution is unclear (Fig. 2i-j). The foramen jugulare posterius is bounded medially by the exoccipitals, and is mostly open laterally, although it is partially bounded anterolaterally by the quadrate (Fig. 2i-j), as in *C. placidoi* and *Foxemys* spp. (Gaffney *et al.*, 2006). On the right side, two unequally-sized foramina nervi hypoglossi are present, the larger of the two positioned dorsolaterally to the smaller (Fig. 2i-j). The basioccipital and basisphenoid are collapsed at the midline, but their margins are still visible, making their overall shape and extent interpretable (Fig. 2e-f). The basioccipital is ovoid in shape, anteroposteriorly short and mediolaterally wide (Fig. 2e-f, i-j). Its contribution to the obscured condylus occipitalis cannot be determined. Slight tuberculae basioccipitale are present (better preserved on the left side), and are small and blunt, bounded by a shallow midline depression as in *C. placidoi*, *Galianemys* spp., and *K. kallamedensis* (Gaffney *et al.*, 2006) (Fig. 2c-f).

Due to the slight quadrate-supraoccipital contact, the prootics do not contact the opisthotics (Fig. 2a-b). There is no evidence of prootic exposure on the ventral surface of the skull, although there is sutural fusion and taphonomic damage in the area. The foramen stapediotemporale is anteriorly positioned on the otic chamber and is formed in the prootic-quadrate suture (Fig. 2g-h). The prootic forms the posterodorsal margin of the foramen nervi trigemini, which is separate from the foramen stapediotemporale as in Cearachelyini and unlike Bothremydini and Taphrosphyini (Gaffney *et al.*, 2006) (Fig. 2g-h).

The opisthotic forms the dorsomedial margin of the fenestra postotica (Fig. 2i-j). It lacks the distinct slit observed in the Cearachelyini. The foramen jugulare posterius is open laterally, making it continuous with the medial aspect of the fenestra postotica, as in Cearachelyini and *Foxemys* spp. (Gaffney *et al.*, 2006) (Fig. 2i-j). The fenestra is almost continuous with the incisura columella auris (Fig. 2i-j), comparable to the condition described in the euraxemydids *Dirqadim* *schaefferi* and *Euraxemys essweini* (Gaffney *et al.*, 2006). The groove for the columella is visible. The basisphenoid is wide and roughly subtriangular, pointing anteriorly (Fig. 2e-f). Medially, it demarcates a moderately sized fossa pterygoidea within which lies the foramen posterior canalis caroticus inferior (fpcci) and foramen nervi ramo vidiani (Fig. 2e-f).

**Carapace**

See Table 1 for shell specimen metrics.

DMNH 2013-07-0683 is a small hexagonal neural with anterolateral sides that are significantly shorter than the posterolateral side (Fig. 3a-d). The edges are finely dentate and there is a small anterior projection at each end of the anterior margin. A slightly wavy transverse sulcus crosses the dorsal surface at the posterior third of the neural, separating vertebral scales 2 and 3 (Fig. 3a-b). The dorsal surface is smooth except for short longitudinal striations along the anterior margin (Fig. 3a). The neural arch articulation on the ventral surface is narrow, ovoid, and elongated near the center of the midline (Fig. 3c-d).

DMNH 2013-07-1998 is the posterior portion of the pygal (Fig. 3e-h). Superficially, the dorsal surface has a worn texture of small, reticulated divots (Fig. 3e). Two slightly curved, relatively wide sulci travel medially from each end to meet at the midline and project directly posterior. These separate bilateral twelfth marginal scales from each other at the midline, and from vertebral scale 5 anteriorly (Fig 3f). The ventral bone surface has similar ornamentation, but only posterior to a thickened ridge that is concave anteriorly (Fig. 3g). The cross section of the posterior margin is acute and its edge is flared slightly dorsally.

DMNH 2013-07-0517 is a left costal 1 that is broken at the axillary buttress and is missing a thin portion of its anterolateral half (Fig. 3i-l). The significantly larger anteroposterior length relative to the width indicates an elongate neural 1, typical of pleurodires (Fig. 3i-l). Fine grooves, perpendicular to the edges, occur around its perimeter on the dorsal surface (Fig. 3i). The grooves converge in the central dorsal surface, where there are small reticular divots (Fig. 3i). Thin sulci separate vertebral scales 1 and 2 from each other in the bone’s medial half and from pleural scale 1 laterally (Fig. 3j). The ventral surface is smooth, and the first rib head is 8.8 mm wide. The rib head has a narrow elliptical articular facet, which is 4.5 mm tall at its posterior edge. The raised rib courses posterolaterally to meet with the posterior lateralmost corner, which is thickened and rough, indicating a probable articulation with the axillary buttress (Fig. 3k-l).

DMNH 2013-07-1999 is a left costal 5 that is missing portions from its anterior middle and lateral quarter (Fig. 3m-p). A gracile rib head projects 1.6 mm from the ventral surface, and depressions flank the neck of the rib anteriorly and posteriorly (Fig. 3o-p). The rib bulging from the ventral surface is approximately 10 mm antero-posteriorly, and it is raised most prominently at its lateral end. The dorsal surface is textured by faint striations perpendicular to the anterior and posterior sutures, and parallel, laterally-oriented grooves course toward the shell margin (Fig. 3m). The anterior and posterior edges are finely sutured, and most of the ventral surface is smooth (Fig. 3o). There are thin, shallow sulci that are nearly indistinguishable from the shell texture on the medial side of the dorsal surface, separating pleural scale 3 from vertebral scales 3 and 4, and vertebral scales 3 and 4 from each other (Fig. 3m-n). However, a fine network of longitudinal grooves surrounds the rib head and neck, covering approximately the medial third of the ventral surface (Fig. 3o). A pair of thin, smooth-edged scratches travel parallel with the posterior edge along most of its ventral surface, indicating possible tooth marks (Fig. 3o-p).

DMNH 2013-07-1320 includes the medial end of a probable right costal 3 (Fig. 3q-t). A finely incised transverse sulcus crosses the dorsal surface and bifurcates near the lateral edge (Fig. 3q-r). The anterior half of the dorsal surface likely belongs to vertebral scale 2, and the posterior to vertebral scale 3 (Fig. 3r). The space between the lateral bifurcation is pleural scale 2 (Fig. 3r). The dorsal surface is covered with faint, longitudinal striations except for some that are oblique near the posterolateral corner (Fig. 3q). On the ventral surface, the rib head and sutural serrations have been taphonomically obliterated (Fig. 3s-t).

DMNH 2013-07-0557 is a probable right costal 3 (Fig. 3u-x). Its dorsal surface is worn, but a faint transverse sulcus is visible, separating likely vertebral scales 2 and 3 (Fig. 3u-v). There is semicircular chip on the medial side of the anterior edge (Fig. 3u-v). The medial edge forms an angle for a probable articulation with neurals 3 and 4 (Fig. 3w-x). Fine sutures are visible but worn along the anterior and posterior edges (Fig. 3u). The ventral surface is worn, and a rib head projects 2.6 mm from the surface (Fig. 3w-x). Its articular facet is approximately rectangular and transversely oriented. The posteromedial border comes to a point which is more prominent ventrally (Fig. w-x).

DMNH 2013-07-1405 is a left costal 6 that was previously attributed to cf. *Algorachelus* sp. (see Adrian *et al.*, 2019) (Fig. 3y-ab). As there is no evidence of more than one pleurodire at the AAS, it is redescribed here and referred to *P. appalachius* gen. et sp. nov. The width is relatively small and the lateral edge slanted, indicating a posterior location among the costals (Fig. 3y-ab). Near the middle of the ventral surface, a shift occurs from relatively smooth bone medially to badly abraded and crushed bone laterally, where the rib and surrounding bone become substantially thicker to meet with bridge peripherals 8 and 9 (Fig. 3aa-ab). A transverse sulcus on the dorsal surface separates pleural scales 3 and 4 (Fig. 3y-z). The sulcus bifurcates at the medial third of the costal to separate the pleural scales from vertebral scale 4. Otherwise, the dorsal surface is covered in the finely incised, vermiculating grooves typical of bothremydids (Fig. 3y).

DMNH 2013-07-0525 is the medial end of a likely left costal 4 (Fig. 3ac-af). On the ventral surface, a rib bulge is prominent and tapers medially to a rib head that projects 3.7 mm (Fig. 3ae-af). The articular facet is worn, and forms a narrow ellipse that extends posterolaterally from the midline (Fig. 3ae-af). The ventral texture is mostly obscured by wear, though a small berm branches from the rib bulge to the posterior edge halfway along its width (Fig. 3ae). The dorsal surface is also worn, as well as the finely dentate anterior and posterior sutures (Fig. 3ac-ad). Faint longitudinal grooves are visible, especially near the anterolateral corner (Fig. 3ac). A histological thin section of DMNH 2013-07-0525 was cut from its lateral edge, and its internal microanatomy is described below.

DMNH 2013-07-1320 also includes a medial portion of a probable right costal 4, which is broken on its lateral aspect medial to the dorsal sulci (Fig. 3ag-aj). The dorsal surface is nearly smooth except for faint longitudinal striations, especially toward its medial side (Fig. 3ag-ah). Its medial edge is obtusely angled for likely articulation with neural 4 anteriorly and neural 5 posteriorly (Fig. 3ag-aj). Fine sutures are visible ventrally along its anterior and posterior edges (Fig. 3ai). Just posterior to mid-length, there is a small rib head with a trapezoidal articular facet (Fig 3ai-aj). It is angled slightly posteriorly, rising approximately 1.7 mm from the ventral surface.

DMNH 2013-07-0673 is a right peripheral 7, forming the posterior end of the bridge (Fig. 3ak-ap). Its lateral margin is 77.8° between the dorsal and ventral surfaces anteriorly, but it becomes more acute (35.3°) posteriorly (Fig. 3ao-ap). Its medial aspect is dominated by the concavity that comprises the inside of the shell, which is tall and deep anteriorly, but compressed and shallow posteriorly (Fig. 3ak-ap). Neither dorsal nor ventral surfaces have a discernable texture, though the anterior half of the ventral surface has three ridges that become shorter laterally (Fig. 3am-an). These are lateral to the pleuromarginal sulcus that runs the length of the peripheral, and anterior to the sulcus between marginal scales 7 and 8 (Fig. 3am-an). The latter sulcus continues to the ventral side of the bone, where it approximately bisects the peripheral (Fig. 3 ak-al). Sutures at the anterior and posterior edges are finely dentate (Fig. 3ak-an), and the shell bone is thicker posteriorly.

DMNH 2013-07-1279 is a left peripheral 9 (Fig. 3aq-at). It is uniformly flat, except slightly upturned laterally, and it is missing its posterolateral corner (Fig. 3aq-at). Its dorsal surface has a fine, but worn texture of tiny reticular pits and grooves and parallel striations perpendicular to the medial suture (Fig. 3aq). Sulci cross the dorsal surface, separating marginal scales 9 and 10 from each other on the lateral side, and from pleural scales 3 and 4 medially (Fig. 3aq-ar).

**Postcrania**

DMNH 2013-07-0500 is an isolated proximal left humerus, broken near the proximal diaphysis (Fig. 4a-g). The specimen has a maximum anteroposterior length of 18.9 mm, and is 35.0 mm mediolaterally. The humeral head is hemispherical and is tilted slightly laterally at approximately 105° from the diaphysis (Fig. 4a). The head measures 14.4 mm tall, 12.2 mm wide, and is approximately 6.2 mm deep. A small tuberosity projects on the proximal ventral portion of the head (Fig. 4c). The medial process is nearly as tall as the head, and slightly taller than the lateral process (Fig. 4a-d). The medial process has a maximum width of 8.8 mm and projects 18.6 mm from the humeral head (Fig. 4a). It forms an approximately 63° angle with the dorsoventral axis when viewed dorsally (Fig. 4e). The lateral process reaches a width of 9.4 mm and projects 13.6 mm at approximately 42° from the anteroposterior axis. The proximal portion of the lateral process forms a wide shoulder between the lateralmost edge and the humeral head (Fig. 4a, c). In ventral view, the proximal edge of the lateral process slopes gently downward, but this area on the opposite side is concave proximally, forming a shallow saddle (Fig. 4c). A “C”-shaped intertubercular fossa lies between the two processes, which is similar in shape to other pleurodires (Fig. 4c).

**4. Histological Description**

We sampled a histological thin section from DMNH 2013-07-0525, the medial portion of a partial left costal 4 (Fig. 5). The slice was cut from the lateral edge of the specimen, capturing the rib bulge on the ventral surface (Fig. 5a). The bone histology of *P. appalachius* gen. et sp. nov. is similar to that of *Bothremys barberi*, *Taphrosphys sulcatus*, *Foxemys mechinorum*, and *Cearachelys placidoi* with an asymmetrical diploë build in the shell bone (Scheyer, 2007; Sena *et al.*, 2020). The external cortex (ECO) of *P. appalachius* is approximately 1.5 times the thickness of the internal cortex (ICO) (Fig. 5a). The ECO is composed of compact bone near the external surface, and it contains several poorly defined lamellae, parallel to the external surface (Fig. 5b). The lamellae are suggestive of faint growth marks, which are best preserved in the neurals of bothremydids (Scheyer, 2007). Nearby sequences of Sharpey’s fibers are oriented subperpendicular to the external surface (Fig. 5b). Diagonal collagenous fibers bundles are incorporated into all layers of the external cortices, and become nearly perpendicular to the bone surface in the externalmost layers (Fig. 5b). The external cortex is moderately vascularized near the anterior and posterior sutures, but less so near the center of the bone (Fig. 5a). Vascular spaces are flattened with long axes parallel to the external surface of the bone (Fig. 5a). The more interior zone of the external cortex is formed by interwoven structural fibers (ISF), which are diagonally arranged and collagenous (Fig. 5b), as in *Bothremys barberi*, *Taphrosphys sulcatus*, and *Foxemys mechinorum* (Scheyer, 2007). Lacunae are common in the ISF between intersecting collagen fiber bundles (Fig. 5a). In the cancellous bone near the anterior and posterior sutured edges, the trabeculae are arranged in rows between parallel lamellae that lose their organization as they transition into the center of the bone comprising the rib (Fig. 5a). The interstitial trabeculae of the interior cancellous bone are surrounded by lamellar bone (Fig. 5c). Cancellous bone trabeculae are short, thick, and rounder near the interior of the bone, but dorso-ventrally flattened toward the external and internal cortices (Fig. 5a). Some trabeculae are rectangular with rounded corners and arranged in series that fill irregular radial compartments, oriented toward the interior of the rib (Fig. 5a, c). There are scattered secondary osteons in the cancellous bone (Fig. 5b). Lacunae are round in the woven bone and flattened in the nearby parallel-fibered bone, and the transition from the interior cancellous bone to the parallel fibered bone of the internal cortex is poorly defined (Fig. 5d).

**5. Character State Analysis**

Character state optimization revealed that the *P. appalachius* gen. et sp. nov. branch is characterized by two character state changes: For character 40, the postorbital is smaller than the orbit; for character 128, the basisphenoid is more triangular. One of the unique features of *P. appalachius* gen. et sp. nov. is its extremely open incisura columella auris (ica) that is confluent with the fenestra postotica (Fig. 2g-j). An open ica is also found in in euraxemydids, and a few other bothremydids: *Cearachelys*, *Foxemys,* and *Polysternon* (Gaffney et al., 2006).

Three other taxa are also positioned at the base of Bothremydidae. *Sankuchemys sethnai* has one character state change, presence of a maxillary accessory ridge (character 60). *Kurmademys kallamedensis* has two character state changes relating to the position of the foramen posterius carotici canali internus (fpcci) restricted to the basisphenoid (character 99) outside the pterygoid (character 101). As in Hermanson et al. (2020), *Kinkonychelys rogersi* has no character state changes on its branch. Three character state changes defined Bothremydidae in all trees: extensive contact between the quadrate and exoccipital (character 114), condylus mandibularis that is wider than long (character 89) and separated from the cavum tympani region (character 90). The clade of Cearachelyini + Bothremydini + Taphrosphyini is not defined by any character state changes, but each tribe therein contains its own set of character state changes. The Bothremydodda (Bothremydini + Taphrosphyini) is defined by eight character state changes.

**6. Additional References**

Adams, R. L. & Carr, J. P. Regional depositional systems of the Woodbine, Eagle Ford, and Tuscaloosa of the U. S. Gulf Coast. *Gulf Coast Association of Geological Societies Transactions* **60**, 3-27 (2010).

Adams, T. L., Polcyn, M. J., Mateus, O., Winkler, D. A. & Jacobs, L. L. First occurrence of the long-snouted crocodyliform *Terminonaris* (Pholidosauridae) from the Woodbine Formation (Cenomanian) of Texas. *Journal of Vertebrate Paleontology* **31**, 712-716, doi:10.1080/02724634.2011.572938 (2011).

Adams, T. L., Noto, C. R. & Drumheller, S. A large neosuchian crocodyliform from the Upper Cretaceous (Cenomanian) Woodbine Formation of North Texas. *Journal of Vertebrate Paleontology* **37**, e1349776, doi:10.1080/02724634.2017.1349776 (2017).

Adrian, B., Smith, H. F., Noto, C. R. & Grossman, A. A new baenid, "Trinitichelys" maini sp. nov., and other fossil turtles from the Upper Cretaceous Arlington Archosaur Site (Woodbine Formation, Cenomanian), Texas, USA. *Palaeontologia Electronica* **22.3.81**, 1-29, doi:10.26879/1001 (2019).

Bennett, G., Main, D., Anderson, K. & Peterson, R. in *Geological Society of America Abstracts with Programs.*

Broin, F. d. Les tortues et le Gondwana. Examen des rapports entre le fractionnement du Gondwana et la dispersion géographique des tortues pleurodires à partir du Crétacé. *Stvdia Geologica Salamanticensia: Stvdia Palaeocheloniologica* **2**, 103-142 (1988).

D'Amore, D. C. & Blumenschine, R. J. Komodo monitor (*Varanus komodoensis*) feeding behavior and dental function reflected through tooth marks on bone surfaces, and the application to ziphodont paleobiology. *Paleobiology* **35**, 525-552 (2009).

Dilcher, D. & Crane, P. *Archaenthus*: An early angiosperm from the Cenomanian of the Western Interior of North America. *Annals of the Missouri Botanical Garden* **71**, 351-383, doi:10.2307/2399030 (1984).

Gaffney, E. S., Campos, D. d. A. & Hirayama, R. *Cearachelys,* a new side-necked turtle (Pelomedusoides: Bothremydidae) from the Early Cretaceous of Brazil. *American Museum Novitates* **3319**, 1-20 (2001).

Gaffney, E. S., Tong, H. & Meylan, P. A. *Galianemys*, a new side-necked turtle (Pelomedusoides: Bothremydidae) from the Late Cretaceous of Morocco. *American Museum Novitates* **3379**, 1-20 (2002).

Gaffney, E. S., Tong, H. & Meylan, P. A. Evolution of the side-necked turtles: the families Bothremydidae, Euraxemydidae, and Araripemydidae. *Bulletin of the American Museum of Natural History* **300**, 1-318, doi:10.1206/0003-0090(2006)300[1:EOTSTT]2.0.CO;2 (2006).

Gaffney, E. S. *et al.* *Acleistochelys*, a new side-necked turtle (Pelomedusoides: Bothremydidae) from the Paleocene of Mali. *American Museum Novitates* **3549**, 1-24 (2007).

Joyce, W. G., Lyson, T. R. & Kirkland, J. I. An early bothremydid (Testudines, Pleurodira) from the Late Cretaceous (Cenomanian) of Utah, North America. *PeerJ* **4:e2502** doi:10.7717/peerj.2502 (2016).

Kennedy, W. J. & Cobban, W. A. Cenomanian ammonite faunas from the Woodbine Formation and lower part of the Eagle Ford Group, Texas. *Palaeontology* **33**, 75-154 (1990).

Krause, D. W. & Baird, D. Late Cretaceous mammals east of the North American Western Interior Seaway. *Journal of Paleontology* **53**, 562-565 (1979).

Lapparent de Broin, F. & Werner, C. New late Cretaceous turtles from the Western Desert, Egypt. *Annales de Paléontologie* **84**, 131-214, doi:10.1016/S0753-3969(98)80005-0 (1998).

Lee, Y.-N. The Archosauria from the Woodbine Formation (Cenomanian) in Texas. *Journal of Paleontology* **71**, 1147-1156, doi:10.1017/S0022336000036088 (1997a).

Lee, Y.-N. Bird and dinosaur footprints in the Woodbine Formation (Cenomanian), Texas. *Cretaceous Research* **18**, 849-864, doi:10.1006/cres.1997.0091 (1997b).

MacNeal, D. L. *The flora of the Upper Cretaceous Woodbine Sand in Denton County, Texas*. Vol. 19 (1958).

Main, D. J. *Paleoenvironments and Paleoecology of the Cenomanian Woodbine Formation of Texas: Paleobiogeography of the Hadrosaurs (Dinosauria: Ornithischia)* Master of Science thesis, University of Texas, Arlington, (2005).

Main, D. J. *Appalachian delta plain paleoecology of the Cretaceous Woodbine Formation at the Arlington Archosaur Site, North Texas* PhD thesis, The University of Texas, (2013).

Main, D. J., Parris, D. C., Grandstaff, B. G. & Carter, B. A new lungfish (Dipnoi: Ceratodontidae) from the Cretaceous Woodbine Formation, Arlington Archosaur Site, North Texas. *Texas Journal of Science* **63**, 283-298 (2011).

Main, D. J., Noto, C. R. & Weishampel, D. B. in *Hadrosaurs* (eds D.A. Eberth & D.C. Evans) Ch. 5, 77-95 (Indiana University Press, 2014).

Njau, J. K. & Blumenschine, R. J. A diagnosis of crocodile feeding traces on larger mammal bone, with fossil examples from the Plio-Pleistocene Olduvai Basin, Tanzania. *Journal of Human Evolution* **50**, 142-162 (2006).

Noto, C. R. in *Society of Vertebrate Paleontology Annual Meeting* (ed Christopher R. Noto) 38-51 (2015).

Noto, C. R. New theropods from the Woodbine Formation of Texas: insights into Cenomanian Appalachian Ecosystems. *Journal of Vertebrate Paleontology, Program and Abstracts* **197** (2016).

Noto, C. R., Main, D. J. & Drumheller, S. K. Feeding traces and paleobiology of a Cretaceous (Cenomanian) crocodyliform: example from the Woodbine Formation of Texas. *Palaios* **27**, 105-115, doi:10.2110/palo.2011.p11-052r (2012).

Noto, C. R., Drumheller, S., Adams, T. L. & Turner, A. H. An enigmatic small neosuchian crocodyliform from the Woodbine Formation of Texas. *The Anatomical Record*, doi:10.1002/ar.24174 (2019).

Oliver, W. B. Depositional systems in the Woodbine Formation (Upper Cretaceous), northeast Texas: The University of Texas at Austin. *Bureau of Economic Geology Report of Investigations* **73**, 28 (1971).

Pérez-García, A. New information on the Cenomanian bothremydid turtle *Algorachelus* based on new, well-preserved material from Spain. *Fossil Record* **21**, 119-135 (2018).

Pérez-García, A. *et al.* A bothremydid from the middle Cenomanian of Portugal identified as one of the oldest pleurodiran turtles in Laurasia. *Cretaceous Research* **78**, 61-70, doi:10.1016/j.cretres.2017.05.031 (2017).

Scheyer, T. M. *Comparative bone histology of the turtle shell (carapace and plastron): implications for turtle systematics, functional morphology and turtle origins* PhD thesis, University of Bonn, (2007).

Sena, M. V. d. A., Bantim, R. A. M., Saraiva, A. Á. F., Sayão, J. M. & Oliveira, G. R. Osteohistology and microanatomy of a new specimen of *Cearachelys placidoi* (Testudines: Pleurodira) a side-necked turtle from the Lower Cretaceous of Brazil. (2020).

Stephenson, L. W. & Stenzel, H. B. Larger invertebrate fossils of the Woodbine Formation (Cenomanian) of Texas, with decapod crustraceans from the Woodbine Formation of Texas *USGS Professional Paper* **242**, 1-225, doi:10.3133/pp242 (1952).

Trudel, P. *Stratigraphic sequences and facies architecture of the Woodbine-Eagle Ford interval, Upper Cretaceous, North Central Texas* Unpublished Masters thesis, Tarleton State University, (1994).

Winkler, D., Jacobs, L., Lee, Y. & Murry, P. in *Sixth Symposium on Mesozoic Terrestrial Ecosystems and Biota* *Short Papers* (eds A. Sun & Y. Wang) (China Ocean Press, 1995).

**7. Tables**

**Table 1**. Measurements in mm of shell elements included in this study.

| Specimen (DMNH 2013-07-) | Max. length | Max. width | Max. thickness |
| --- | --- | --- | --- |
| 0683 | 16.6 | 13.0 | 5.0 |
| 1998 | 22.1 | 35.2 | 4.6 |
| 0517 | 55.3 | 78.0 | 8.8 |
| 1999 | 32.1 | 107.5 | 7.3 |
| 1320 | 27.4 | 25.1 | 7.1 |
| 0557 | 19.8 | 18.1 | 5.0 |
| 1405 | 31.6 | 84.1 | 18.7 |
| 0525 | 24.4 | 29.5 | 6.7 |
| 1320 | 20.8 | 22.7 | 5.7 |
| 0673 | 24.6 | 20.7 | 9.73 (ant.), 17.1 (post.) |
| 1279 | 17.0 | 21.2 | 2.8 |
